# Discovery of Novel UV-Filters with Favorable Safety Profiles in the 5-Arylideneimidazolidine-2,4-dione Derivatives Group

**DOI:** 10.3390/molecules24122321

**Published:** 2019-06-24

**Authors:** Justyna Popiół, Agnieszka Gunia-Krzyżak, Kamil Piska, Dorota Żelaszczyk, Paulina Koczurkiewicz, Karolina Słoczyńska, Katarzyna Wójcik-Pszczoła, Anna Krupa, Agata Kryczyk-Poprawa, Ewa Żesławska, Wojciech Nitek, Paweł Żmudzki, Henryk Marona, Elżbieta Pękala

**Affiliations:** 1 Department of Pharmaceutical Biochemistry, Faculty of Pharmacy, Jagiellonian University Medical College, Medyczna 9, 30-688 Krakow, Poland; justyna.popiol@uj.edu.pl (J.P.); kamil.piska@uj.edu.pl (K.P.); paulina.koczurkiewicz@uj.edu.pl (P.K.); karolina.sloczynska@uj.edu.pl (K.S.); katarzynaanna.wojcik@uj.edu.pl (K.W.-P.); elzbieta.pekala@uj.edu.pl (E.P.); 2 Department of Bioorganic Chemistry, Chair of Organic Chemistry, Faculty of Pharmacy, Jagiellonian University Medical College, Medyczna 9, 30-688 Krakow, Poland; dorota.zelaszczyk@uj.edu.pl (D.Z.); henryk.marona@uj.edu.pl (H.M.); 3 Department of Pharmaceutical Technology and Biopharmaceutics, Faculty of Pharmacy, Jagiellonian University Medical College, Medyczna 9, 30-688 Krakow, Poland; a.krupa@uj.edu.pl; 4 Department of Inorganic and Analytical Chemistry, Faculty of Pharmacy, Jagiellonian University Medical College, Medyczna 9, 30-688 Krakow, Poland; agata.kryczyk@uj.edu.pl; 5 Department of Chemistry, Institute of Biology, Pedagogical University of Cracow, Podchorążych 2, 30-084 Krakow, Poland; ewa.zeslawska@up.krakow.pl; 6 Faculty of Chemistry, Jagiellonian University, Gronostajowa 2, 30-387 Krakow, Poland; wojciech.nitek@uj.edu.pl; 7 Department of Medicinal Chemistry, Faculty of Pharmacy, Jagiellonian University Medical College, Medyczna 9, 30-688 Krakow, Poland; pawel.zmudzki@uj.edu.pl

**Keywords:** UV-filters, UV radiation, 5-arylidenehydantoin derivatives, photoprotection, safety evaluation

## Abstract

Effective protection from the harmful effects of UV radiation may be achieved by using sunscreens containing organic or inorganic UV filters. The number of currently available UV filters is limited and some of the allowed molecules possess limitations such as systemic absorption, endocrine disruption properties, contact and photocontact allergy induction, and low photostability. In the search for new organic UV filters we designed and synthesized a series consisting of 5-benzylidene and 5-(3-phenylprop-2-en-1-ylidene)imidazolidine-2,4-dione (hydantoin) derivatives. The photoprotective activity of the tested compounds was confirmed in methanol solutions and macrogol formulations. The most promising compounds possessed similar UV protection parameter values as selected commercially available UV filters. The compound diethyl 2,2′-((*Z*)-4-((*E*)-3-(4-methoxyphenyl)allylidene)-2,5-dioxoimidazolidine-1,3-diyl)diacetate (**4g**) was characterized as an especially efficient UVA photoprotective agent with a UVA PF of 6.83 ± 0.05 and favorable photostability. Diethyl 2,2′-((*Z*)-4-(4-methoxybenzylidene)-2,5-dioxo- imidazolidine-1,3-diyl)diacetate (**3b**) was the most promising UVB-filter, with a SPF*_in vitro_* of 3.07 ± 0.04 and very good solubility and photostability. The main photodegradation products were geometric isomers of the parent compounds. These compounds were also shown to be non-cytotoxic at concentrations up to 50 µM when tested on three types of human skin cells and possess no estrogenic activity, according to the results of a MCF-7 breast cancer model.

## 1. Introduction

Sunscreens are necessary to protect the skin against the acute and chronic consequences of ultraviolet radiation (UVR). The main active ingredients of sunscreens called UV-filters are inorganic blockers which reflect or scatter UVR or organic absorbers. Organic UV-filters act as chemical energy converters—after absorption of photons the molecule is excited to a higher energy state, then the absorbed energy is dissipated by the emission of photons or heat and molecule returns to the ground state [1,2].

In the European Union sunscreen products are classified as cosmetics, but in the USA, Australia and Canada they are recognized as over-the-counter (OTC) drugs, thus requirements for their safety and efficacy are comparable with those of other dermatological drugs [3,4]. Modern organic UV-filter molecules are expected to be not only UV-absorbers with high extinction coefficients, but they must also be photostable, safe and readily soluble in cosmetics solvents. It is estimated that process of commercialization of a new UV-filter takes about 10 years [5,6]. Typical exposure to UV-filters is very high because of their concentration in sunscreens which reaches 10% for some organic UV-filters. Moreover, the product is applied on the entire surface of the skin. Some UV-filters or their metabolites were detected in the blood [7], urine [8,9] and milk of nursing mothers [10], what indicates that these compounds penetrate into the bloodstream. More concerns appeared after several adverse effects of currently used UV-filters were recognized. Many of them may cause endocrine disruption [11,12,13] and adversely affect the viability of nerve cells, which may contribute to the development of neurodegenerative diseases [14]. Additionally, benzophenone-3 showed cytogenetic effect on human lymphocytes increasing micronuclei and chromosomal aberrations frequencies [15]. The other limitations of currently available UV-filters are related to contact and photocontact allergy induction [16,17] and unsatisfactory photostability [18,19]. One of the most popular UVB-filter, octinoxate (INCI: ethylhexyl methoxycinnamate, EHMC), upon ultraviolet irradiation undergoes a photoisomerisation process, and the resulting *Z*-isomer has a lower extinction coefficient, which results in a significant reduction in its photoprotectiveness [20]. Moreover, it was shown that the photolysis products of EHMC are more toxic to the mammalian cells than EHMC alone. Low photostablity also characterises an UVA-filter—avobenzone (INCI: butyl methoxy-dibenzoylmethane). After irradiation its ability to absorb UVA is significantly reduced, additionally, it was shown that arylglyoxals, the main products of avobenzone photodegradation, are strong sensitizers [21]. Due to their unsatisfactory safety profile according to the European Commission regulations, in the latest years two UV-filters—4-aminobenzoic acid (PABA) and 3-benzylidene camphor—have been banned from use in cosmetics [22,23].

Considering the disadvantages of currently available UV-filters, the search for new UV-filters remains an important issue. The list of UV filters allowed in cosmetic products within the European Union is still expanding. In 2014 2,4,6-tris([1,1’-biphenyl]-4-yl)-1,3,5-triazine (INCI: trisbiphenyl triazine) was authorized [24], whereas in 2018 2,2’-methylene-bis-(6-(2*H*-benzotriazol-2-yl)-4-(1,1,3,3-tetramethylbutyl)phenol) (INCI: methylene bisbenzotriazolyltetramethylbutylphenol nano) was added to the list [25]. Moreover, several scientific papers on this topic were published recently. Researchers have focused on modifications of currently used compounds which represent derivatives of benzophenone, dibenzoylomethane, benzotriazole, phenylbenzimidazole and cinnamic acid [26,27,28,29,30].

**Figure 1:**
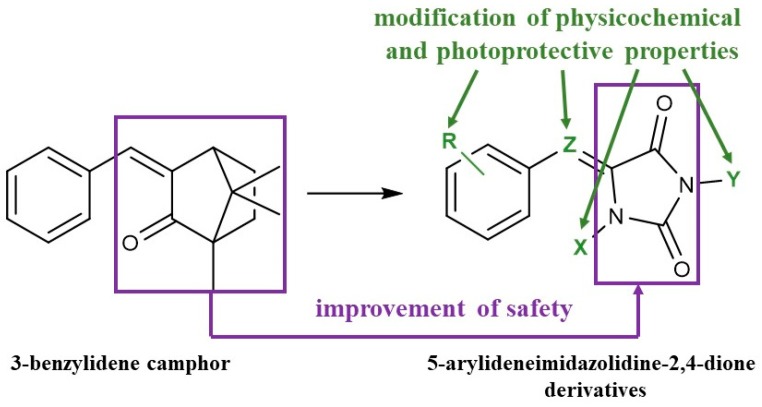
Design concept of the tested series: camphor was exchanged with hydantoin, while the benzylidene moiety remained unchanged or was extended.

Derivatives of 5-arylideneimidazolidine-2,4-dione, depending of the presence and location of the substituents on the phenyl or imidazolidine rings exhibit diverse biological activity. Their ability to modulate cancer efflux pumps and the melanogenesis process, antimicrobial, and antiproliferative activity, as well as α1-adrenoceptor antagonistic properties were reported [31,32,33,34,35]. Their ultraviolet absorbing properties were also described [36,37], which prompted us to synthesize and evaluate the photoprotective activity of a new series of 5-arylideneimidazolidine-2,4-dione (5-arylidenehydantoin) derivatives. For further characterization of the most promising compounds we also performed tests on their photostability and safety. We designed the compounds on the basis of the structure of 3-benzylidenecamphor, in which the camphor fragment was replaced with a hydantoin moiety. We anticipated that this structural change would positively affect the safety profile of the molecules (Figure 1). We also planned several further modifications such as introduction of substituents in the phenyl ring and/or hydantoin fragment as well as extension of the linker by adding an allyl group in order to modify the physicochemical and photoprotective properties of the tested compounds.

## 2. Results and Discussion

### 2.1. Chemistry

**Scheme 1:**
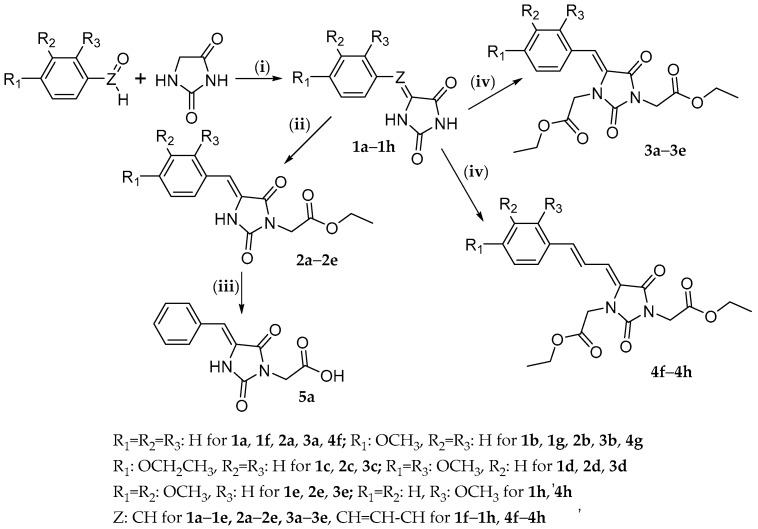
Synthesis and structures of tested compounds. *Reagents and Conditions*: (**i**) Knoevenagel condensation CH_3_COONa/CH_3_COOH; (**ii**) ClCH_2_COOEt, K_2_CO_3_, TEBA, acetone; (**iii**) EtOH/H_2_O, NaOH; (**iv**) BrCH_2_COOEt, K_2_CO_3_, DMF.

Synthesis and chemical structures of the tested compounds are shown in Scheme 1. These compounds were obtained from appropriately substituted benzaldehyde (compounds **1a–1e, 2a–2e, 3a–3e, 5a**) or cinnamaldehyde (compounds **4f–4h**) and hydantoin. 

In the first step, appropriate 5-arylideneimidazolidine-2,4-diones (5-arylidenehydantoins, compounds **1a**–**1h**) were obtained by Knoevenagel condensations. Physicochemical analyses were performed to confirm their structures and purity. Compounds **1a**–**1h** served for further syntheses, while compound **1e** was also tested for its photoprotective properties and safety. Compounds **1a**–**1h** were used in reactions with ethyl chloroacetate or ethyl bromoacatate to synthesize substituted on nitrogen(s) mono- (compounds **2a**–**2e**) or diesters (compounds **3a**–**3e** and **4f**–**4h**), respectively. Additionally, compound **2a** was subjected to hydrolysis in order to obtain the corresponding acid (compound **5a**). Chemical structures and purity of final compounds were confirmed by spectral analysis (NMR, LC/MS/MS).

### 2.2. X-ray Crystallographic Studies

**Figure 2:**
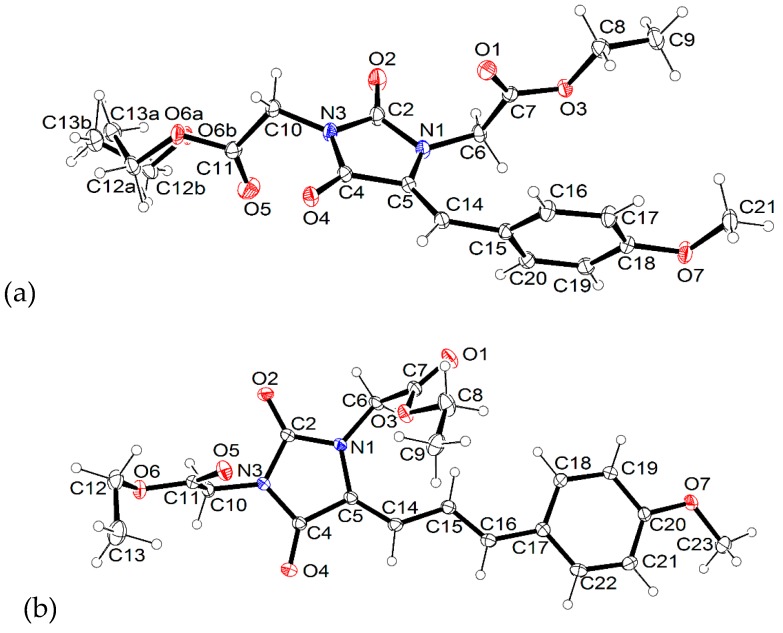
The molecular geometries of (**a**) **3b** and (**b**) **4g**, with the atom numbering schemes. Displacement ellipsoids are drawn at the 30% probability level.

The molecular geometries in the crystal structures of **3b** and **4g**, together with the atom numbering schemes, are shown in Figure 2.

Both molecules contain a double bond between the C5 and C14 atoms. The crystal structures confirm the *Z* arrangement at this bond. The molecule of **4g** possesses an additional double bond between the C15 and C16 atoms, and at this bond the *E* arrangement is observed. The angles between the planes of the hydantoin and aromatic rings are 72.68(4)° and 9.21(7)° for **3b** and **4g**, respectively, which implies that the 5-benzylidenehydantoin fragment of **3b** is not planar, while the 5-cinnamylidenehydantoin fragment of **4g** is almost planar. We have previously determined crystal structures of 5-benzylidene-2-selenohydantoin derivatives, wherein the 5-benzylidene-2-selenohydantoin fragment is almost planar [38,39]. Considering that, we have carried out a search in the Cambridge Structural Database (CSD) [40] for 5-benzylidenehydantoin (20 structures found), 5-benzylidene-2-thiohydantoin (10 structures found) and 5-benzylidene-2-selenohydantoin (two structures found) fragments. Most of the crystal structures deposited in the CSD possess an almost planar benzylidene(thio/seleno)hydantoin moiety (Figure S1). The non-planarity of the benzylidenehydantoin fragment is very seldom observed in crystal structures, while for thio- and seleno-hydantoins it is not observed at all.

The CSD includes no crystal structures containing cynnamylidenehydantoin moiety. We have determined earlier the crystal structures containing similar fragment, namely cinnamylidene rhodanine moiety [41], this fragment was also almost planar like in **4g**.

Both investigated molecules possess the same 2-ethoxy-2-oxoethyl substituents at the nitrogen atoms (N1 and N3). There are some differences in the mutual orientation of these substituents in both molecules (Figure S2). The substituents at the N3 atom differ mainly in conformation of the ethyl group. The C11-O6-C12-C13 torsion angle has values of −101.5(5)° (for occupancy A) in **3b** and 88.0(2)° in **4g**. Significant differences are visible in the conformation of the substituent at the N1 atom. The torsion angle C5-N1-C6-C7 has values of −65.5(1)° and 67.8(2)° for **3b** and **4g**, respectively, what implies opposite orientation of these substituents.

**Figure 3:**
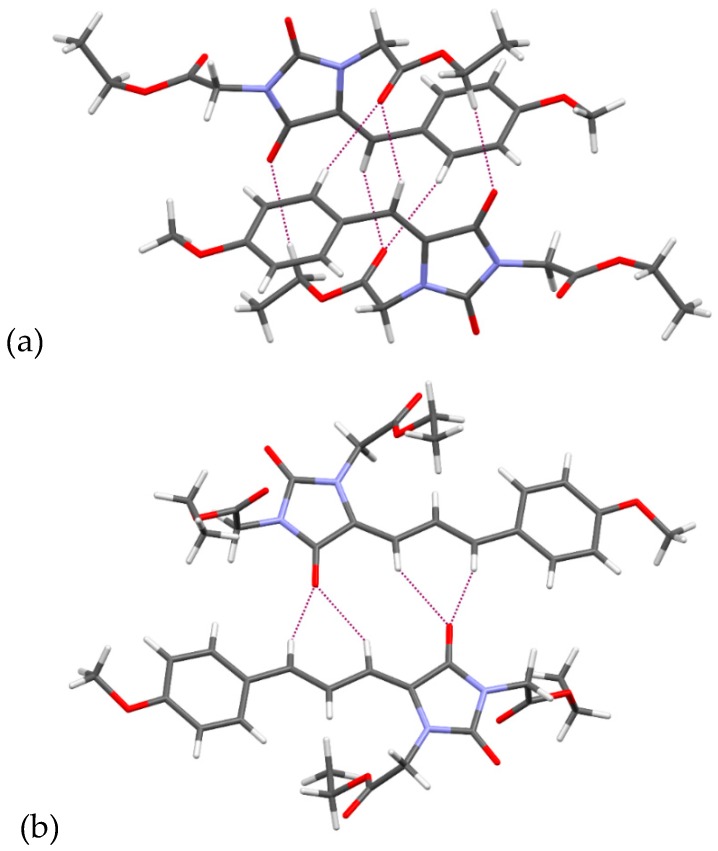
The intermolecular interactions of two molecules of (**a**) **3b** and (**b**) **4g**. Dashed lines indicates hydrogen bonds. The disordered fragment of **3b** is depicted only for major occupancy.

The crystal packing is determined by C-H···O intermolecular interactions (Table S1). In the **3b** two molecules, related by the inversion center, create dimers by C8-H8A···O4, C14-H14···O1 and C16-H16···O1 intermolecular interactions (Figure 3a), while in **4g** the are also observed dimers, but created by other contacts, namely C14-H14···O4 and C16-H16···O4 (Figure 3b).

### 2.3. Ultraviolet Spectroscopic Properties

Among tested compounds there are both UVA and UVB absorbers (λ_max_ from 295 to 379 nm). Their ultraviolet absorbing properties are presented in Table 1 and Figure S3. Compounds **2a**–**2e**, which belong to the group of ethyl esters of (*Z*)-2-(4-benzylidene-2,5-dioxoimidazolidin-1-yl)acetic acid, absorb UV radiation with different range, λ_max_ and ε_max_ depending on the presence and position of the auxochrome. They show higher molar absorption coefficients and wider absorption ranges than commercially used UV filters such as 4-methylbenzylidene camphor (4-MCB), ethylhexyl methoxycinnamate or octocrylene. The presence of an additional *N*-alkyl substituent in the imidazolidine ring in compounds **3b**–**3e** contributes to a hypsochromic shift by 15 nm (in compound **3a** by 21 nm) as well as a hypochromic effect (ε_max_ decreased almost two-fold) when compared to *N*-monosubstituted derivatives (compounds **2a**–**2e**) which is consistent with former findings [42]. Thus *N,N*-bis-substituted derivatives of 5-benzylideneimidazolidine-2,4-dione are slightly weaker UV absorbers than *N*-monosubstituted derivatives, but their ε_max_ still surpass octocrylene. The λ_max_ of compounds **2a** and **3a** without substituents on the phenyl ring is located in the UVB region (316 and 295 nm, respectively), while the λ_max_ of other 5-benzylideneimidazolidine-2,4-dione derivatives with methoxy groups is shifted to the UVA II or UVA I region.

The tested (*Z*)-5-((*E*)-3-phenylallylidene)imidazolidine-2,4-dione derivatives **4f**–**4h** show the highest molar absorption coefficients among the tested series (especially compounds **4f** and **4g**). Elongation of the unsaturated spacer caused a significant bathochromic shift in comparison to the corresponding benzylidene analogues. Their UV absorption ranges and λ_max_ are very similar to those of avobenzone, which is one of the most commonly used UV filters.

### 2.4. Photoprotective Activity

Absorption spectra and thus the photoprotective properties of UV-filters depend on the solvent used [43], hence to investigate the functional absorbing efficacy of the tested compounds and reference UV-filters (octocrylene, EHMC, 4-MBC, and avobenzone) they were tested in macrogol formulations (F1–F3) at 2% (*w/w*) concentration.

The composition of formulations and concentration of tested compounds were based on their limited solubility. *N*-monosubstituted compounds **2a**–**2e** were insoluble at desirable concentrations in the typical solvents used in cosmetic formulations such as alcohols, triacetin, surfactants, and co-surfactants. To dissolve compounds **2a** and **2b** before incorporation to the formulation DMSO: Tween 20 (1:9 *w/w*) was used, for less soluble **2c**–**2e** it was necessary to use DMSO alone as solvent. Incorporation of an additional 2-ethoxy-2-oxoethyl substituent in the imidazolidine ring of *N*-mono-substituted 5-arylidene-2,4-imidazolidinediones significantly improved the solubility of the compounds so it was possible to eliminate DMSO from the formulation and use the solvent system triacetin:Tween 20 (1:9 *w/w*) to dissolve compounds **3a**–**3e** and **4f**–**4h** before incorporation into the formulation. Thus the detailed composition of the formulations is as follows: F1–Tween 20 43.2%, PEG-400 25%, PEG-1500 25%, Triacetin 4.8%, tested compound 2%; F2–Tween 20 43.2%, PEG-400 25%, PEG-1500 25%, DMSO 4.8%, tested compound 2%; F3–PEG-400 25%, PEG-1500 25%, DMSO 48%, tested compound 2%. Considering the disadvantages of currently reported macrogol formulations and the low concentration achieved for the tested compounds (2%), we plan to work on cosmetic emulsions containing the most promising derivatives at higher concentrations as well as required excipients including preservatives, antioxidants, etc. This research will be carried out in the near future.

**Figure 4:**
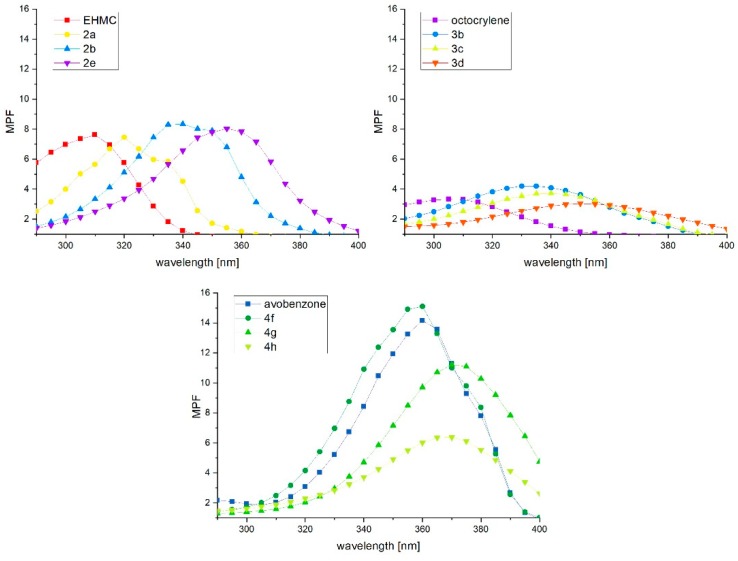
UV absorption spectra of tested compounds and reference UV filters obtained in macrogol formulation at 2% (*w/w*) concentration applied on polymethylmethacrylate plates. MPF–Monochromatic Protection Factor.

The results of studies on the photoprotective activity of the tested compounds are presented in Table 2 and Figure 4. The performed study indicate that all tested compounds can be considered as potential UV-filters. In the group of ethyl esters of (*Z*)-2-(4-benzylidene-2,5-dioxoimidazolidin-1-yl)acetic acid (compounds **2a**–**2e**), compound **2a** shows the highest value of SPF*_in vitro_*, which is comparable to EHMC SPF*_in vitro_*. This is related to the fact that the λ_max_ of **2a** is located in the UVB region. On the other hand, the λ_max_ of compounds **2b**–**2e** is shifted to UVA II (**2b**, **2c**) or UVA I (**2d**, **2e**) region due to presence of electron donating groups in the phenyl ring, thus these compounds besides higher SPF*_in vitro_* than octocrylene, show higher efficiency in UVA absorption (UVA PF from 4.29 ± 0.11 to 4.77 ± 0.24) than **2a** and reference UV-filters excluding avobenzone.

*N*,*N*-bis-substituted 5-benzylideneimidazolidine-2,4-dione derivatives **3a**–**3e** may be characterized as slightly weaker UV-filters. Their SPF*_in vitro_* values range from 1.84 ± 0.02 to 3.07 ± 0.04. The highest values are found for compounds **3a** (2.97 ± 0.45) and **3b** (3.07 ± 0.04), which are lower than for EHMC and 4-MBC, but higher than for octocrylene. Additionally, compounds **3b**–**3e** provides higher UVA protection in comparison to the reference UV-filters excluding avobenzone and range from 2.17 ± 0.11 for **3e** to 2.59 ± 0.23 for **3b**.

Among (*Z*)-5-((*E*)-3-phenylallylidene)imidazolidine-2,4-dione derivatives **4f**–**4g** there are strong potential UVA-filters. Their UVA PF values range from 4.28 ± 0.02 to 8.43 ± 1.35. The best efficacy is found for compounds **4f** and **4g**. The absorption curve of **4f** is very similar to that of avobenzone, thus **4f** seems to be a good candidate as a novel alternative to avobenzone in photoprotective formulations. Compound **4g** shows lower UVA PF, despite higher molar absorption coefficient at its λ_max_ when compared to **4f** and avobenzone, which is probably caused by crystallization of the compound from solvents during formulation. The significantly higher UVA PF values of these compounds in comparison to 5-benzylideneimidazolidine-2,4-dione are associated with the presence of an additional conjugated double bond. The extension of the π-system contributes to the bathochromic shift and higher extinction coefficient, thus UVA or broad spectrum UV-filters have higher molecular weight than UVB filters [43].

The impact of the presence and position of alkoxy groups in the phenyl ring is also visible when another important parameters that characterize sunscreen formulations such as critical wavelength (λ_c_) and UVA/UVB ratio are compared. Compounds like **2a**, **3a**, **4f** without substituents in the phenyl ring show the lowest value of λ_c_ and UVA/UVB ratio in comparison to their analogues with alkoxy groups. These values are increasing when substituents in the 4- (compounds **2b**, **3b**, **4g**) and 3,4- (compounds **2e**, **3e**) positions are present and are the highest when electron donating groups are in the 2,4- position (compounds **2d**, **3d**).

### 2.5. Photostability Studies

#### 2.5.1. Photostability in Methanol Solutions

The photostability of the tested compounds in methanol solutions was investigated by the measure of the changes in the λ_max_ and in the area under curve (AUC) upon irradiation. Results are presented in Table S2 and Figure S4. After irradiation of both mono- and diester 5-(Z)-benzylideneimidalidine-2,4-dione derivatives a bathochromic shift and slight hypochromic effect are observed. In the group of monoesters with methoxy substituents on the phenyl ring (compounds **2b**–**2e**) λ_max_ shifts from 5 to 8 nm and decreases of the area under the curve from 0.96 to 4.33% are found. In the case of compound **2a** without a phenyl ring substituent, only a 1 nm shift of λ_max_ and almost a 30% decrease of AUC are found. After irradiation of diester derivatives **3a**–**3e**, bathochromic shifts from 6 to 11 nm are observed. The photostability of 3-phenylallylidene derivatives **4f**–**4h** is reduced in comparison to the benzylidene derivatives substituted on the phenyl ring (compounds **2b**–**2e**, **3b**–**3e**). Although no shifts are observed, the decrease of AUC reaches to 40.9% in the case of the non-substituted derivative **4f**. For compounds **4g** and **4h** with methoxy groups in the *para* or *ortho* position, the decrease of AUC is 19.9 and 1.5%, respectively. In comparison to the reference UV-filters, the tested compounds, excluding **4f,** show better photostability than EHMC, for which a 36.7% decrease of AUC is detected.

The analysis of chromatograms and mass spectra of selected compounds obtained after appropriate irradiation suggests that changes in absorption curves result from photoisomerisation processes taking place in solution, which are a very efficient way of dispersing the absorbed energy that affects many UV filters, e.g., cinnamic acid derivatives and benzylidene camphor derivatives [44] (Figure S5). In post-irradiation chromatograms the additional peak with the same mass as tested compound appears which indicates that the observed photoproduct is a geometric isomer. The percentage of photoisomer formation range from 22.02 to 36.03%. In general, the tested compounds may be regarded as photostable in methanol solutions.

#### 2.5.2. Photostability in Cosmetic Formulations 

**Figure 5:**
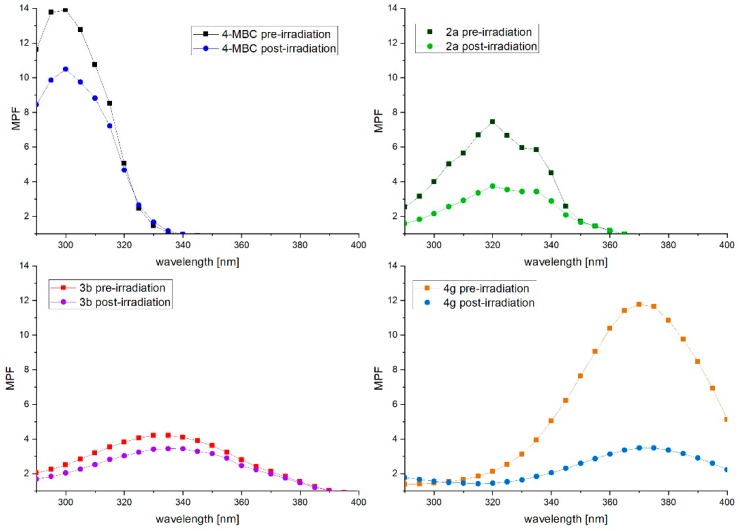
UV absorption spectra of tested compounds and 4-MBC pre-irradiation and after 1 hour irradiation with solar light simulator conducted at 500 W/m^2^ obtained in 2% (*w/w*) macrogol formulations.

After irradiation of thin layer of formulations applied on PMMA plates, their photoprotective parameters were determined and compared with the pre-irradiation results. The study gives an overview on functional photostability of the tested compounds. It is an important test, because the photostability of some compounds in low concentrated solutions may meaningfully differ from results obtained for higher concentrations used in sunscreen products [45]. According to the previous studies, formulations whose SPF do not decrease more than 20% after irradiation are considered photostable [46,47]. In the present study both UVB and UVA-absorbers were tested, so the changes in SPF*_in vitro_*, UVA PF and AUC after irradiation are taken into account as a measure of photostability. The tested were performed for formulations described in photoprotective activity section, the results are presented in Table 3 and Figure 5.

Compounds **3a**–**3e** in the macrogol formulation show the highest photostability–they may be considered as photostable UV-absorbers. The decrease of SPF*_in vitro_* from 10.1 to 19.5% was observed. The percentage of initial UVA PF and AUC values range from 103.5 to 87.0% and 94.0 to 79.6% respectively. Especially compound **3d** is characterized by functional photostability very similar to that of 4-MBC which is considered a photostable UV-filter [48]. The analysis of chromatograms and mass spectra of selected compounds indicates that in macrogol formulations the compounds behave as in methanol solutions. In the post-irradiation chromatogram of compound **3b**, 32.1% of photoisomer is observed while 28.4% is detected in methanol solution. Additionally, a new photoproduct with 2.4% relative peak area appears. Similarly, in the post-irradiation chromatogram of compound **3c**, a photoproduct with 2.8% relative peak area is detected. In the chromatogram of compound **3d** no additional peaks besides the photoisomer are found.

Interesting results are obtained for compounds **2a**–**2e**. These compounds are insoluble in the solvent system selected for compounds **3a**–**3e**, thus it was necessary to dissolve them in DMSO before incorporation into the formulation. The functional photostability of these compounds is drastically reduced in comparison to the results obtained in methanol solutions. The decrease of tested parameters is from 41.1% for **2d** and reaches 75.9% for **2b**. This was probably due to evaporation of the solvent from the formulation and thus the precipitation of the compound. To confirm that the reason of the low functional photostability of compounds **2a**–**2e** was their precipitation, additional tests for compounds **2a** and **2b** in formulations containing a reduced amount of DMSO (from 48 to 4.8%) were carried out. The results indicates an improvement of the functional photostability by 31.1% for **2a** and by 30.2% for **2b**. Additionally, the analysis of post-irradiation chromatograms of **2a** shows that despite significant changes in the reduction of photoprotection parameters between two formulations differing in the content of DMSO, the percentage of photoisomer formation is very similar for both formulations (45% vs 48%). These findings encourage us to search for another, better solvent system for compounds **2a**–**2e**.

Photostability evaluation of compounds **4f**–**4h** in macrogel formulations indicates that as in methanol solutions they are not photostable. The decrease of their AUC was from 30.8% for **4h** and reached 72.1% for **4f**. In the corresponding post-irradiation chromatograms, beside photoisomers, the peaks of photodegradation products are also detected. The percentage of photoproducts content ranges from 11.0% for **4h** to 22.9% for **4f**. This result does not discredit compounds **4f**–**4h**. Low photostability is also associated with other UV-filters, namely avobenzone and ethylhexyl methoxycinnamate, but it can be improved by adding, for example, triplet-state quenchers to the formulations [20,49].

There are many factors that contribute to the photostability of a potential UV-filter molecule. These compounds are from different chemical groups, their photobehaviour depends on the structure and presence of functional groups. High photostability is a feature of molecules with intramolecular hydrogen bonds such as hydroxyphenyltriazine derivatives. In this class of compounds after absorption of a photon a tautomeric transfer of a H-atom to an N-atom is observed and then the absorbed energy is lost as thermal energy (vibronic relaxation) [6]. Compounds containing unsaturated bonds (such as derivatives of 5-arylidenehydantoin, camphor and cinnamic acid) after absorbing a photon undergo a photoisomerisation process. This chemical transformation is a desirable way to lose absorbed energy. It is most preferably when the *cis* and *tans* forms have similar absorption curve shapes and extinction coefficients but forming a photoisomer often results in a significantly lower absorption intensity [20].

### 2.6. Preliminary Safety Assessment

The safety profile of selected compounds (**1e**, **2d**, **2e**, **3b**, **4g**, **4f** and **5a**) was evaluated. A cytotoxicity assay in a unique skin panel model, containing three types of cells that are involved in the structural organization of human skin was performed. Additionally, considering the possibility of absorption of the compounds into the bloodstream and reports about adverse effects on viability of nerve cells caused by some UV-filters [14], cytotoxicity against hepatocytes (HepG2) and neuroblastoma cell lines (SH-SY5Y) was evaluated. As reference standards 4-MBC and/or BP-1 were used. A MTT (3-(4,5-dimethylthiazol-2-yl)-2,5-diphenyltetrazolium bromide) assay was used to investigate metabolic activity of cells incubated in the presence of the analyzed compounds. Estrogenic activity of compounds was also determined, where the UV-filter benzophenone-2 (BP-2) exhibiting estrogenic activity was used as a positive control. Our preliminary safety assessment of the compounds also included evaluation of their metabolic stability. Skin not only is the major protective environmental barrier, but also is involved in the biosynthesis of endogenous compounds, detoxification of xenobiotics and also participates in the activation of pro-toxic substances [50]. As a consequence, metabolism studies are becoming a consideration in the development of new cosmetic ingredients. The xenobiotic metabolising enzymes within the epidermis in general represent the same classes as liver, although their levels are lower and activities are weaker than those found in liver [51]. On the other hand xenobiotics such as some cosmetic ingredients can enter into systemic circulation through the epidermis and dermis being exposed to liver enzymes [52]. Therefore, we used rodents liver microsomes in this study to assess the rate of stability of selected potential sunscreens. Additionally the mutagenic activity of two selected compounds (**3b**, **4g**) was examined.

#### 2.6.1. Skin Panel Model

**Figure 6:**
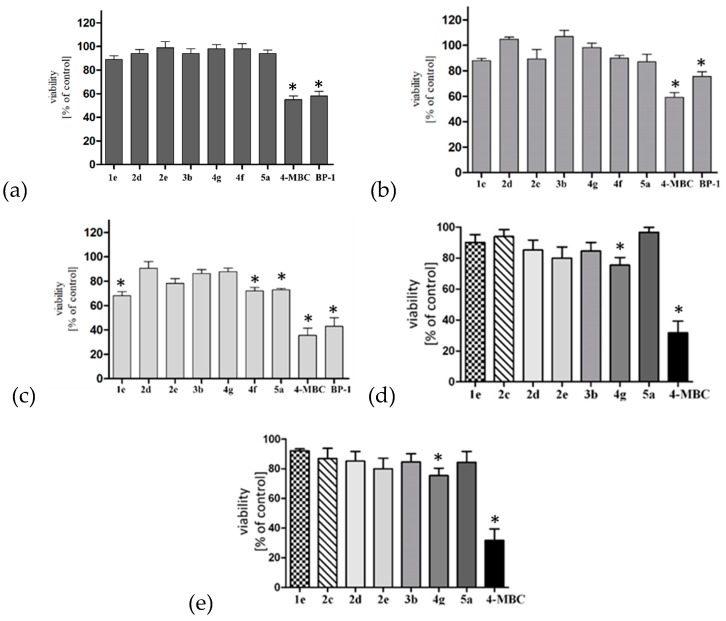
Viability of human keratinocytes HaCaT (**a**), human skin fibroblast (BJ) (**b**) cell lines, human primary dermal melanocytes (**c**), HepG2 cells (**d**) and SH-SY5Y cells (**e**) incubated in the presence of compounds at 50 µM for 24 hours. Cell viability was determined using MTT assay. Each experiment was repeated in triplicate. Graphs represent the number of viable cells expressed as percent of control (cells not treated with compounds) ± SEM (*p* < 0.05).

The cytotoxicity was examined after 24 h incubation of the cells with compounds. Results after incubation at the higher administrated concentration are presented in Figure 6. Cytotoxicity studies on human keratinocytes and human skin fibroblasts showed that all tested compounds except the references 4-MBC and BP-1 are deprived of cytotoxic activity in the range of tested concentrations (2–50 μM), and the viability of BJ cells and HaCaT is ≥87% and 89%, respectively (Figure 6a,b). Cytotoxicity studies on human primary dermal melanocytes indicate a cell viability ≥68% at the highest tested concentration except for BP-1 and 4-MCB, where the viability was <43% and 36% respectively (Figure 6c).

#### 2.6.2. Hepatocytotoxicity and Neurocytotoxicity Studies

Results after incubation of HepG2 cells and SH-SY5Y cells with the tested compounds and reference compound 4-MBC at the highest (50 µM) concentration are presented in Figure 6. Cytotoxicity studies on HepG2 hepatocellular tumor cells indicate that the tested 5-arylidene imidazolidine-2,4-dione derivatives are safe at the concentrations used. In the MTT test, the viability of HepG2 cells at the highest concentration is >75%. For reference compounds used in the test, the observed viability of cells was <32%. Cytotoxicity studies on SH-SY5Y cells indicate the viability of cells >75% at the highest tested concentration with exclusion of 4-MCB, where viability was <31% (Figure 6e).

#### 2.6.3. Estrogenic Activity

**Figure 7:**
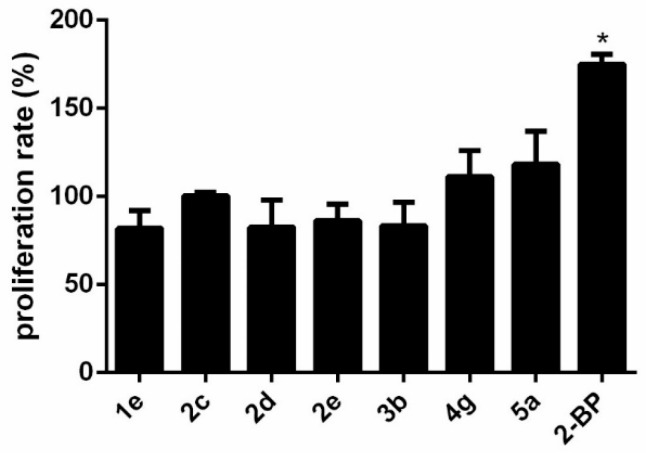
Proliferation rate of MCF-7 cells incubated with tested compounds at 5 µM. Compounds showed lack of proliferation-stimulating effect. BP-2 increased MCF-7 proliferation by over 60% (*p* < 0.05).

Proliferation analyses indicated lack of stimulation effect of tested compounds in MCF-7 breast cancer model. In contrast, BP-2 stimulated proliferation by over 60%, what was statistically significant. Hence, lack of estrogenic activity of compounds was concluded. Results are presented in Figure 7.

#### 2.6.4. In Vitro Stability of Compounds in Mouse Liver Microsomes

Stability and direction of biotransformation of hydantoin derivatives in mouse liver microsomes depend on the structure of the side chains. The metabolic stability was evaluated by determining the half-life (t_1/2_) and intrinsic clearance (Cl_int_) of the compounds upon incubation (Table 4). In the group of studied structures the most stable compound was **5a** (((*Z*)-2-(4-benzylidene-2,5-dioxoimidazolidin-1-yl)acetic acid)) with t_1/2_ higher than 23 hours and the value of in vitro intrinsic clearance Cl_int_ below 1 µL/mg/min. The least stable compound was proved to be **4g** (diethyl 2,2′-((*Z*)-4-((*E*)-3-(4-methoxyphenyl)allylidene)-2,5-dioxoimidazolidine-1,3-diyl)diacetate) with a half-life of 3 min and value of Cl_int_ over 300 µL/mg/min. Other compounds’ stability remained at a moderate level, most of them being slightly less stable than the reference BP-2.

We observed two directions of hydantoin derivatives’ microsomal biotransformation (Table 4). One is *O*-dealkylation of compounds with alkoxy substituents on the phenyl ring (**1e**, **2c**–**e, 3b** and **4g**) while the second is ester hydrolysis of diester derivatives **3b** and **4g** to monoesters. 

Microsomal cytochrome P450 enzymes catalyze the oxidative *O*-dealkylation of xenobiotics, cleaving methoxy or ethoxy groups in aromatic rings vulnerable to this reaction [53,54]. This reaction requires the presence of NADPH. We observed *O*-dealkylation as a biotransformation reaction in case of all tested structures bearing the methoxy (**1e**, **2d, 2e, 3b** and **4g**) or ethoxy (**2c**) side chains. Such results are consistent with the previous reports regarding the biotransformation of structurally similar compounds [53,55]. Apart from CYP450 isoforms other drug metabolizing enzymes are also present in microsomal preparations including carboxylesterases [56] which mainly catalyze the hydrolysis of diverse endogenous and xenobiotic esters. Carboxylesterases (CEs) are present in microsomal fractions of various tissues, including liver, intestine and skin [57]. CEs play crucial roles in the metabolism of various ester xenobiotics including drugs [56] and cosmetic ingredients such as parabens [58,59], or salicylates [60]. On the contrary to CYP450 enzymes no catalytic cofactors are involved in the CEs-catalyzed reactions [59]. Therefore the interpretation of CEs contribution to microsomal stability of compounds requires using additional blank incubations deprived not only NADPH regenerating systems but also microsomes [59]. In our study all tested esters of hydantoin(di)acetic acid derivatives (**2c**–**2e, 3b, 4g**) were stable in buffer at pH 7.4 in the absence of microsome solution showing no sign of spontaneous hydrolysis after 60 min incubation at 37 °C (data not showed). Hydantoin derivative 1,3-diacetate diesters (**3b** and **4g**) were hydrolysed to monoesters (parent compound losing 28 Da, Table 4) in the presence of microsomes and independently of the presence of a NADPH-regenerating system. The hydantoin 3-acetate monoesters derivatives **2c**–**2e** did not hydrolyzed under the same conditions. Therefore the most probable reaction is formation of 3-acetate monoesters from **3b** and **4g** by mouse liver CEs. Formation of monoester from diester by mammalian liver microsomal CEs was recently reported for di(2-ethylhexyl) phthalate [61].

#### 2.6.5. Mutagenic Activity

In the present study an Ames MPFTM Penta 2 microplate format mutagenicity assay (Xenometrix, Allschwil, Switzerland) was used to evaluate mutagenicity of two selected compounds (**3b** and **4g**) exhibiting the most promising photoprotective potential. Growth, exposure and indicator media, liver S9 fraction, S9-NADP, S9-G-6-P, positive control chemicals as well as *Salmonella typhimurium* and *Escherichia coli* strains were included in the kit from Xenometrix. According to the obtained results, there were no doses of test compounds **3b** and **4g** with more than a 2-fold induction over the baseline, and a dose dependent response was not observed both in the absence and presence of metabolic activation (Table S3). Therefore, compounds **3b** and **4g** were clearly non-mutagenic in the absence and presence of metabolic activation.

### 2.7. Structure-Activity Relationship Studies

Monoethyl esters of (*Z*)-2-(4-benzylidene-2,5-dioxoimidazolidin-1-yl)acetic acid (*N*-mono substituted derivatives, compounds **2a**–**2e**) absorbed UV radiation in methanol solutions in the range of 290–394 nm with λ_max_ 316–349 nm depending on the presence and position of the auxochrome. An additional *N*-alkyl substituent in imidazolidine ring in compounds **3b**–**3e** caused a hypsochromic shift by 15–21 nm. Introduction of methoxy group(s) in the phenyl ring caused a shift of λ_max_ from the UVB region for compounds **2a** and **3a** to the UVA region for **2b**–**2e** and **3b**–**3e**. Elongation of the unsaturated spacer between the phenyl and hydantoin rings in compounds **4f**–**4h** contributed to the significant bathochromic shift when compared to the appropriate benzylidene analogues leading to the extension of the UV absorption range into the UVA region. These compounds were also characterized with the highest molar absorption coefficients among the tested series.

The tested compounds, especially **2a**–**2e**, were difficult to dissolve in the proposed macrogol formulations. In order to obtain a 2% formulation it was necessary to use DMSO because common cosmetic solvents were not able to dissolve the compounds. However, we noticed that an additional 2-ethoxy-2-oxoethyl substituent in the imidazolidine ring (as in compounds **3a**–**3e**) caused a significant improvement of the solubility so that the use of DMSO in those formulations was not necessary.

Tests performed in formulations proved that compounds without methoxy substituents in the phenyl ring were characterized as UVB filters while the presence of some methoxy groups shifted the UV-protection properties into the UVA region which was observed as an increase of the UVA PF value. Moreover, compounds without a substituent in the phenyl ring showed the lowest values of critical wavelength (λ_c_) and UVA/UVB ratio in comparison to their analogues possessing alkoxy groups. Values of λ_c_ and UVA/UVB ratio increased with the following kind of substitution in the phenyl ring: no substitution < 4-methoxy < 3,4-dimethoxy < 2,4-dimethoxy.

The currently reported compounds were shown to be less cytotoxic than commercially available UV-filters at concentrations up to 50 µM in tested cell lines (human epidermal keratinocytes HaCaT, human skin fibroblasts BJ, human primary epidermal melanocytes, human hepatoma cells HepG2, human neuroblastoma cell line SH-SY5Y). The results suggest that exchange of camphor with hydantoin, as presented in our design concept (Figure 1) caused an improvement of the safety profile.

## 3. Materials and Methods

### 3.1. Chemistry

2-Methoxycinnamalaldehyde, 4-methoxycinnamalaldehyde, ethyl chloroacetate and ethyl bromoacetate were purchased from Sigma–Aldrich Chemie (Darmstadt, Germany), hydantoin was obtained from TCI Chemicals (Tokyo, Japan), TEBA, 3,4-dimethoxybenzaldehyde and cinnamalaldehyde were purchased from Alfa Aesar (Kandel, Germany), 4-ethoxybenzaldehyde were purchased from Acros Organics (Geel, Belgium) whereas the reagents benzaldehyde, 4-methoxybenzaldehyde and 2,4-dimethoxybenzaldehyde were provided by Fluoro Chem (Hadfield, England). Triacetin was obtained from ICN Biomedicals, Inc. (Irvine, CA, USA), PEG-400 and PEG-1500 were purchased from Merck (Darmstadt, Germany). Solvents were commercially available materials of reagent grade. Melting points (mp) were uncorrected and were determined using a Buchi SMP-20 apparatus (Buchi Labortechnik, Flawil, Switzerland). The ^1^H-NMR (300 MHz) were obtained in CDCl_3_ with a Varian Mercury-VX 300 NMR spectrometer (Varian Inc., Palo Alto, CA, USA) using TMS as an internal standard. Spectral data includes chemical shifts in ppm, multiplicities, constant couplings in Hz, number of protons, protons’ positions. Multiplicities are abbreviated as follow: s (singlet), bs (broad singlet), d (doublet), dd (doublet of doublets), ddd (double doublet of doublets), t (triplet), m (multiplet). The LC/MS system consisted of an Acquity UPLC system (Waters Corporation, Milford, MA, USA) coupled to a Waters TQD mass spectrometer (electrospray ionization mode ESI-tandem quadrupole). All the LC/MS analyses were carried out using an Acquity UPLC BEH C18, 1.7μm, 2.1 × 100 mm column. A flow rate of 0.3 ml/min and a gradient of (5–95)% B over 10 min and then 100% B over 2 min was used. Eluent A: water/0.1% HCO_2_H; eluent B: acetonitrile/0.1% HCOOH. LC/MS data were obtained by scanning the first quadrupole in 0.5 s in a mass range from 50 to 1000 Da; 8 scans were added to produce the final spectrum.

#### 3.1.1. Preparation of (Z)-5-benzylidene- and (Z)-5-((E)-3-phenylallylidene)imidazolidine-2,4-diones (compounds 1a-1h)

Starting materials and compound **1e** were synthesized by means of previously published procedures [62]. Glacial acetic acid (100 mL) was mixed with anhydrous sodium acetate (33.3 g) in round-bottom flask. Then hydantoin (10.1 g, 0.11 mole) was added and mixture was heated under reflux. After achieving boiling point, the appropriate aldehyde (0.1 mole) was added and the mixture was heated for the next 4–8 h. After cooling the mixture was filtered and the obtained solid was washed well with water. Dried crude product was crystallized from ethanol or ethanol/acetic acid (1:1).

#### 3.1.2. Preparation of Ethyl (Z)-2-(4-benzylidene-2,5-dioxoimidazolidin-1-yl)acetates (compounds **2a**–**2e**)

The appropriate (*Z*)-5-beznylideneimidazolidine-2,4-dione (0.01 mole) was mixed with acetone (100 mL) and potassium carbonate (4 g). Then TEBA (0.3 g, 0.001 mole) was added and the mixture was heated under reflux with stirring. To the mixture ethyl chloroacetate (1.22 g, 0.01 M) in acetone (40 mL) was added dropwise and the mixture was heated for 4 h. Then the reaction mixture was filtered, the filtrate was evaporated under reduced pressure and the obtained crude product was recrystallized from ethanol.

#### 3.1.3. Preparation of Diethyl 2,2′-((Z)-4-benzylidene-2,5-dioxoimidazolidine-1,3-diyl) Diacetates and Diethyl 2,2′-((Z)-2,4-dioxo-5-((E)-3-phenylallylidene)imidazolidine-1,3-diyl)diacetates (compounds 3a-3e, 4f-4h)

The appropriate (*Z*)-5-beznylideneimidazolidine-2,4-dione or (*Z*)-5-((*E*)-3-phenylallylidene)-imidazolidine-2,4-dione (0.01 mole) was mixed with potassium carbonate (8 g), then DMF (30 mL) was added. After dissolving the imidazolidine-2,4-dione derivative, ethyl bromoacetate (0.02 M, 3.34 g) was added dropwise. The mixture was stirred at room temperature, while the progress of a reaction was monitored by TLC (chloroform:acetone:methanol 5:1:0.1). After about 8 h the mixture was poured into ice. Obtained solid was collected by filtration. After drying crude product was crystallized from ethanol. 

#### 3.1.4. Preparation of (*Z*)-2-(4-benzylidene-2,5-dioxoimidazolidin-1-yl)acetic acid (**5a**)

Ethyl (*Z*)-2-(4-benzylidene-2,5-dioxoimidazolidin-1-yl)acetate (0.01 M) was mixed with ethanol (50 mL) then NaOH (0.4 g, 0.01 M) dissolved in distilled water was added. The mixture was heated on a water bath. After hydrolysis the solvent was distilled off. The obtained oily residue was dissolved in distilled water then it was treated with 16% sulfuric acid with the aim of separating the free acid. The crude product was crystallized from 50% ethanol.

#### 3.1.5. Physicochemical Properties of Tested Compounds

*(Z)-5-Benzylideneimidazolidine-2,4-dione* (**1a**) was obtained as yellow solid (yield 74%), M = 188.19, mp 222–224 °C, ESI-MS (*m/z*): [M + H]^+^ calcd. for C_10_H_8_N_2_O_2_, 189.19, found, 189.01 100%. Physicochemical data for **1a** were previously published by Martínez-López et al. [63] CAS: 3775-01-7, CAS for *Z* isomer: 74805-60-0.

*(Z)-5-(4-Methoxybenzylidene)imidazolidine-2,4-dione* (**1b**) was obtained as yellow solid (yield 72%), M = 218.21, mp 251–252 °C, ESI-MS (*m/z*): [M + H]^+^ calcd. for C_11_H_10_N_2_O_3_, 219.21, found, 219.11 100%. Physicochemical data for **1b** were previously published by Luo et al. [64] CAS: 5349-42-8, CAS for *Z* isomer: 108402-52-4.

*(Z)-5-(4-Ethoxybenzylidene)imidazolidine-2,4-dione* (**1c**) was obtained as yellow solid (yield 81%), M = 232.24, mp 229–231 °C, ESI-MS (*m/z*): [M+H]^+^ calcd. for C_12_H_12_N_2_O_3_, 233.24, found, 233.13 97.83%. Physicochemical data for **1c** were previously published by Thenmozhiyal et al. [65] CAS: 6325-66-2, CAS for *Z* isomer: none.

*(Z)-5-(2,4-Dimethoxybenzylidene)imidazolidine-2,4-dione* (**1d**) was obtained as yellow solid (yield 82%), M = 248.24, mp 238–240 °C, ESI-MS (*m/z*): [M + H]^+^ calcd. for C_12_H_12_N_2_O_4_, 249.24, found, 249.15 100%. Physicochemical data for **1d** were previously published by Thenmozhiyal et al. [65] CAS: 91559-39-6, CAS for *Z* isomer: 1312438-16-6.

*(Z)-5-(3,4-Dimethoxybenzylidene)imidazolidine-2,4-dione* (**1e**) was obtained as yellow solid (yield 69%), M = 248.42, mp 279–280 °C, ESI-MS (*m/z*): [M + H]^+^ calcd. for C_12_H_12_N_2_O_4_, 249.42, found, 249.08 100%. Physicochemical data for **1e** were previously published by Luo et al. [64] CAS: 10040-91-2, CAS for *Z* isomer: 140894-76-4.

*(Z)-5-((E)-3-Phenylallylidene)imidazolidine-2,4-dione* (**1f**) was obtained as dark yellow solid (yield 73%), M = 214.22, mp 272–274 °C, ^1^H-NMR (DMSO-d_6_) δ 11.09 (s, 1H, -NH-), 10.66 (s, 1H, -NH-), 7.54–7.44 (m, 2H, Ar-H3, Ar-H5), 7.44–7.20 (m, 4H, =CH-, Ar-H2, Ar-H4, Ar-H6), 6.86 (dd, *J* = 29.2, 15.7 Hz, 1H, -CH=), 6.23 (dd, *J* = 11.9, 0.8 Hz, 1H, -CH=), ESI-MS (*m/z*): [M + H]^+^ calcd. for C_12_H_10_N_2_O_2_, 215.12, found, 215.13 100%. Physicochemical data for **1f** were previously published by Lamiri et al. [66] CAS: 66835-63-0, CAS for *Z,E* isomer: 137920-57-1.

*(Z)-5-((E)-3-(4-Methoxyphenyl)allylidene)imidazolidine-2,4-dione* (**1g**) was obtained as brown solid (yield 82%), M = 244.25, mp 289–291 °C, ^1^H-NMR (DMSO-d_6_) δ 11.02 (s, 1H, -NH-), 10.57 (s, 1H, -NH-), 7.43 (d, *J* = 8.7 Hz, 2H, Ar-H2, Ar-H6), 7.11 (dd, *J* = 15.5, 11.8 Hz, 1H, =CH-), 6.95 (d, *J* = 8.8 Hz, 2H, Ar-H3, Ar-H5), 6.86 (d, *J* = 15.5 Hz, 1H, -CH=), 6.21 (dd, *J* = 11.8, 0.7 Hz, 1H, -CH=), 3.76 (s, 3H, O-CH_3_), ESI-MS (*m/z*): [M + H]^+^ calcd. for C_13_H_12_N_2_O_3_, 245.25, found, 245.16 100%. CAS: 664353-78-0, CAS for *Z,E* isomer: none.

*(Z)-5-((E)-3-(2-Methoxyphenyl)allylidene)imidazolidine-2,4-dione* (**1h**) was obtained as brown solid (yield 64%), M = 244.25, mp 260–263 °C, ^1^H-NMR (DMSO-d_6_) δ 11.05 (s, 1H, -NH-), 10.65 (s, 1H, -NH-), 7.55 (dd, *J* = 7.8, 1.7 Hz, 1H, Ar-H6), 7.33–7.16 (m, 2H, Ar-H4, -CH=C), 7.10 (d, *J* = 15.7 Hz, 1H, -CH=), 7.05–6.90 (m, 2H, Ar-H3, Ar-H5), 6.24 (d, *J* = 11.5 Hz, 1H, =CH-), 3.82 (s, 3H, -OCH3), ESI-MS (*m/z*): [M + H]^+^ calcd. for C_13_H_12_N_2_O_3_, 245.25, found, 245.10 98.85%. CAS for *E*,*E* isomer: 1776969-29-9, CAS for *Z*,*E* isomer: none.

*Ethyl (Z)-2-(4-benzylidene-2,5-dioxoimidazolidin-1-yl)acetate* (**2a**) was obtained as white solid (yield 63%) M = 274.28, mp 169–171 °C, ^1^H-NMR (chloroform-d) δ 8.55 (s, 1H, -NH), 7.49–7.44 (m, 1H, Ar-H4), 7.44–7.30 (m, 4H, Ar-H2, Ar-H3, Ar-H5, Ar-H6), 6.79 (s, 1H, -CH=), 4.37 (s, 2H, -CH_2_-), 4.24 (q, *J* = 7.2 Hz, 2H, -CH_2_-), 1.35–1.18 (m, 3H, -CH_3_), ESI-MS (*m/z*): [M + H]^+^ calcd. for C_14_H_14_N_2_O_4_, 275.28, found, 275.20 96.84% CAS: 463317-70-6 CAS for *Z* isomer: 1416719-77-1.

*Ethyl (Z)-2-(4-(4-methoxybenzylidene)-2,5-dioxoimidazolidin-1-yl)acetate* (**2b**) was obtained as white solid (yield 52%), M = 304.30, mp 178–179 °C, ^1^H-NMR (chloroform-d) δ 8.34 (s, 1H, -NH), 7.45–7.34 (m, 2H, Ar-H2, Ar-H6), 7.01–6.90 (m, 2H, Ar-H3, Ar-H5), 6.76 (s, 1H, -CH=), 4.37 (s, 2H, N-CH_2_), 4.24 (q, *J* = 7.1 Hz, 2H, -CH_2_-), 3.85 (s, 3H, -OCH_3_), 1.29 (t, *J* = 7.1 Hz, 3H, -CH_3_) ESI-MS (*m/z*): [M + H]^+^ calcd. for C_15_H_16_N_2_O_5_, 305.30, found, 305.17 99,31%. CAS: 463317-69-3 CAS for *Z* isomer: 2247638-41-9.

*Ethyl (Z)-2-(4-(4-ethoxybenzylidene)-2,5-dioxoimidazolidin-1-yl)acetate* (**2c**) was obtained as white solid (yield 61%), M = 318.33, mp 181–182 °C, ^1^H-NMR (chloroform-d) δ 8.66–8.60 (m, 1H, -NH), 7.48–7.34 (m, 2H, Ar-H2, Ar-H6), 6.98–6.87 (m, 2H, Ar-H3, Ar-H5), 6.75 (s, 1H, -CH=), 4.37 (s, 2H, N-CH_2_), 4.23 (q, *J* = 7.2 Hz, 2H, -CH_2_-), 4.07 (q, *J* = 7.0 Hz, 2H, -CH_2_-), 1.43 (t, *J* = 7.0 Hz, 3H, -CH_3_), 1.36–1.19 (m, 3H, -CH_3_) ESI-MS (*m/z*): [M + H]^+^ calcd. for C_16_H_18_N_2_O_5_, 319.33, found, 319.20 99.02%.

*Ethyl (Z)-2-(4-(2,4-dimethoxybenzylidene)-2,5-dioxoimidazolidin-1-yl)acetate* (**2d**) was obtained as light yellow solid (yield 56%), M = 334.33, mp 197–198 °C, ^1^H-NMR (chloroform-*d*) δ 7.98 (s, 1H, -NH), 7.31–7.21 (m, 1H, Ar-H6), 6.75–6.68 (m, 1H, -CH=), 6.61–6.47 (m, 2H, Ar-H3, Ar-H5), 4.34 (s, 2H, -CH_2_-), 4.23 (q, *J* = 7.1 Hz, 2H, -CH_2_-), 3.93 (s, 3H, -OCH_3_), 3.85 (s, 3H, -OCH_3_), 1.34–1.21 (m, 3H, -CH3) ESI-MS (*m/z*): [M + H]^+^ calcd. for C_16_H_18_N_2_O_6_, 335.33, found, 335.37 100%.

*Ethyl (Z)-2-(4-(3,4-dimethoxybenzylidene)-2,5-dioxoimidazolidin-1-yl)acetate* (**2e**) was obtained as white solid (yield 48%), M = 334.33, mp 198–200 °C, ^1^H-NMR (chloroform-d) δ 8.41 (s, 1H, -NH), 7.10–7.00 (m, 1H, Ar-H6), 6.97–6.87 (m, 2H, Ar-H2, Ar-H5), 6.75 (s, 1H, -CH=), 4.36 (s, 2H, N-CH_2_), 4.23 (q, *J* = 7.1 Hz, 2H, -CH_2_-), 3.93 (s, 3H, 3-OCH_3_), 3.92 (s, 3H, 4-OCH_3_), 1.28 (t, *J* = 7.2 Hz, 3H, -CH_3_), ESI-MS (*m/z*): [M + H]^+^ calcd. for C_16_H_18_N_2_O_6_, 335.33, found, 335.15 100%. **CAS**: 463317-68-2 **CAS** for *Z* isomer: none.

*Diethyl 2,2′-((Z)-4-benzylidene-2,5-dioxoimidazolidine-1,3-diyl)diacetate* (**3a**) was obtained as white solid (yield 81%), M = 360.37, mp 47–49 °C, ^1^H-NMR (chloroform-d) δ 7.39–7.30 (m, 3H, Ar-H3, Ar-H4, Ar-H5), 7.26–7.19 (m, 2H, Ar-H2, Ar-H6), 7.01 (s, 1H, -CH_2_=), 4.37 (s, 2H, -CH_2_-), 4.23 (q, *J* = 7.2 Hz, 2H, -CH_2_-), 4.20 (s, 2H, -CH_2_-), 3.95 (q, *J* = 7.1 Hz, 2H, -CH_2_-), 1.28 (t, *J* = 7.2 Hz, 3H, -CH_3_), 1.09 (t, *J* = 7.1 Hz, 3H, -CH_3_). ESI-MS (*m/z*): [M + H]^+^ calcd. for C_18_H_20_N_2_O_6_, 361.37, found, 361.27 96.74%.

*Diethyl 2,2′-((Z)-4-(4-methoxybenzylidene)-2,5-dioxoimidazolidine-1,3-diyl)diacetate* (**3b**) was obtained as white solid (yield 79%), M = 390.39, mp 74–76 °C, ^1^H-NMR (chloroform-d) δ 7.24–7.13 (m, 2H, Ar-H2, Ar-H6), 6.98 (s, 1H, -CH=), 6.95–6.84 (m, 2H, Ar-H3, Ar-H5), 4.38 (s, 2H, -CH_2_-), 4.30–4.18 (m, 4H, -CH_2_-, -CH_2_-), 4.01 (q, *J* = 7.1 Hz, 2H, -CH_2_-), 3.83 (s, 3H, O-CH_3_), 1.30 (t, *J* = 7.2 Hz, 3H, -CH_3_), 1.12 (t, *J* = 7.1 Hz, 3H, -CH_3_), ESI-MS (*m/z*): [M + H]^+^ calcd. for C_19_H_22_N_2_O_7_, 391.39, found, 391.17 100%.

*Diethyl 2,2′-((Z)-4-(4-ethoxybenzylidene)-2,5-dioxoimidazolidine-1,3-diyl)diacetate* (**3c**) was obtained as light yellow solid (yield 84%), M = 404.42, mp 44–46 °C, ^1^H-NMR (chloroform-*d*) δ 7.22–7.11 (m, 2H, Ar-H2, Ar-H6), 6.97 (s, 1H, -CH=), 6.95–6.82 (m, 2H, Ar-H3, Ar-H5), 4.37 (s, 2H, N1-CH_2_), 4.30–4.15 (m, 4H, N3-CH_2_, -CH_2_-), 4.02 (dq, *J* = 12.6, 7.1 Hz, 4H, -CH_2_-, -CH_2_-), 1.42 (t, *J* = 7.0 Hz, 3H, -CH_3_), 1.34–1.21 (m, 3H, -CH_3_), 1.17–1.04 (m, 3H, -CH_3_), ESI-MS (*m/z*): [M + H]^+^ calcd. for C_20_H_24_N_2_O_7_, 405.42, found, 405.26 100%.

*Diethyl 2,2′-((Z)-4-(2,4-dimethoxybenzylidene)-2,5-dioxoimidazolidine-1,3-diyl)diacetate* (**3d**) was obtained as white solid (yield 75%), M = 420.42, mp 88–90 °C, ^1^H-NMR (chloroform-d) δ 7.05 (dt, *J* = 8.1, 0.7 Hz, 1H, Ar-H6), 6.94 (s, 1H, -CH=), 6.54–6.41 (m, 2H, Ar-H3, Ar-H5), 4.36 (s, 2H, -CH_2_-), 4.29–4.16 (m, 4H, -CH_2_-, -CH_2_-), 4.00 (qd, *J* = 7.1, 0.6 Hz, 2H, -CH_2_-), 3.82 (s, 3H, 4-OCH_3_), 3.79 (s, 3H, 2-OCH_3_), 1.34–1.21 (m, 3H, -CH_3_), 1.12 (t, *J* = 7.1, 0.6 Hz, 3H, -CH_3_), ESI-MS (*m/z*): [M + H]^+^ calcd. for C_20_H_24_N_2_O_8_, 421.42, found, 421.22 100%.

*Diethyl 2,2′-((Z)-4-(3,4-dimethoxybenzylidene)-2,5-dioxoimidazolidine-1,3-diyl)diacetate* (**3e**) was obtained as light yellow solid (yield 68%), M = 420.42, mp 80–82 °C, ^1^H-NMR (chloroform-d) δ 6.97 (s, 1H, -CH=), 6.90–6.78 (m, 2H, Ar-H5, Ar-H6), 6.76 (d, *J* = 1.8 Hz, 1H, Ar-H2), 4.38 (s, 2H, -CH_2_-), 4.32–4.16 (m, 4H, -CH_2_-,-CH_2_-), 4.01 (qd, *J* = 7.2, 0.6 Hz, 2H, -CH_2_-), 3.90 (s, 3H, -OCH_3_), 3.86 (s, 3H, -OCH_3_), 1.33–1.25 (m, 3H, -CH_3_), 1.18–1.05 (m, 3H, -CH_3_). ESI-MS (*m/z*): [M + H]^+^ calcd. for C_20_H_24_N_2_O_8_, 421.42, found, 421.22 100%.

*Diethyl 2,2′-((Z)-2,4-dioxo-5-((E)-3-phenylallylidene)imidazolidyne-1,3-diyl)diacetate* (**4f**) was obtained as yellow solid (yield 84%), M = 386.40, mp 99–100 °C, ^1^H-NMR (DMSO-d_6_) δ 7.57–7.49 (m, 2H, Ar-H2, Ar-H6), 7.49–7.25 (m, 3H, Ar-H3, Ar-H4, Ar-H5), 7.18–7.00 (m, 2H, -CH=, -CH-), 6.66–6.53 (m, 1H, =CH-), 4.92 (s, 2H, -CH_2_-), 4.35 (d, *J* = 4.1 Hz, 2H, -CH_2_-), 4.22–4.06 (m, 4H, -CH_2_-, -CH_2_-), 1.26–0.98 (m, 6H, -CH_3_, -CH_3_), ESI-MS (*m/z*): [M + H]^+^ calcd. for C_20_H_22_N_2_O_6_, 387.40, found, 387.19 100%.

*Diethyl 2,2′-((Z)-4-((E)-3-(4-methoxyphenyl)allylidene)-2,5-dioxoimidazolidine-1,3-diyl)diacetate* (**4g**) was obtained as yellow solid (yield 89%), M = 416.43, mp 134–135 °C, ^1^H-NMR (DMSO-d_6_) δ 7.55–7.42 (m, 2H, Ar-H2, Ar-H6), 7.12–6.86 (m, 4H, -CH=, =CH-, Ar-H3, Ar-H5), 6.55 (d, *J* = 11.9 Hz, 1H, -CH=), 4.90 (s, 2H, -CH_2_-), 4.35 (s, 2H, -CH_2_-), 4.13 (qd, *J* = 7.1, 3.7 Hz, 4H, -CH_2_-, -CH_2_-), 3.77 (s, 3H, O-CH_3_), 1.19 (t, *J* = 7.1 Hz, 3H, -CH_3_), 1.13 (t, *J* = 7.1 Hz, 3H, -CH_3_). ESI-MS (*m/z*): [M + H]^+^ calcd. for C_21_H_24_N_2_O_7_, 417.43, found,417.16 100%.

*Diethyl 2,2′-((Z)-4-((E)-3-(2-methoxyphenyl)allylidene)-2,5-dioxoimidazolidine-1,3-diyl)diacetate* (**4h**) was obtained as yellow solid (yield 75%), M = 416.43, mp 134–135 °C, ^1^H-NMR (chloroform-d) δ 7.38 (dd, *J* = 7.7, 1.7 Hz, 1H, Ar-H6), 7.36–7.18 (m, 2H, -CH=, -CH=), 7.17–7.03 (m, 1H, Ar-H4), 7.00–6.75 (m, 2H, =CH-, Ar-H5), 6.69 (dd, *J* = 11.0, 0.6 Hz, 1H, Ar-H3), 4.72 (s, 2H, -CH_2_-), 4.36 (s, 2H, -CH_2_-), 4.24 (qd, *J* = 7.2, 6.1 Hz, 4H, -CH_2_-, -CH_2_-), 3.89 (s, 3H, 2-OCH_3_), 1.36–1.11 (m, 6H, -CH_3_, -CH_3_), ESI-MS (*m/z*): [M + H]^+^ calcd. for C_21_H_24_N_2_O_7_, 417.43, found, 417.29 97.93%.

*(Z)-2-(4-Benzylidene-2,5-dioxoimidazolidin-1-yl)acetic acid* (**5a**) was obtained as white solid (yield 49%), M = 246.22, mp 215–217 °C, ^1^H-NMR (DMSO-*d*_6_) δ 10.91 (s, 1H, -OH), 7.69–7.59 (m, 2H, -NH, Ar-H4), 7.47–7.28 (m, 4H, Ar-H1, Ar-H2, Ar-H5, Ar-H6), 6.58 (s, 1H, -CH=), 4.19 (s, 2H, -CH_2_-)., ESI-MS (*m/z*): [M + H]^+^ calcd. for C_12_H_10_N_2_O_4_, 247.22, found, 247.16 100%. CAS: 857796-52-2 CAS for *Z* isomer: 850636-92-9.

### 3.2. Crystal Structures of ***3b*** and ***4g***

Crystals suitable for an X-ray structure analysis were obtained from ethanol by slow evaporation of the solvent at room temperature. Data for single crystals were collected using the Oxford Diffraction SuperNova four circle diffractometer (Agilent, Wroclaw, Poland), equipped with the Mo (0.71073 Å) Kα radiation source and graphite monochromator. The phase problems were solved by direct methods using SIR-2014 program [67]. All non-hydrogen atoms were refined anisotropically using weighted full-matrix least-squares on F2. Refinement and further calculations were carried out using SHELXL program [68]. The hydrogen atoms bonded to carbons were included in the structure at idealized positions and were refined using a riding model with Uiso(H) fixed at 1.2 Ueq of C and 1.5 Ueq for methyl groups. The fragment O6-C12-C13 in **3b** is disordered. The occupancy factors of these atoms after refinement are 0.797 and 0.203 for the major (A) and minor (B) components, respectively. For molecular graphics ORTEP [69] and MERCURY [70] programs were used.

**3b**: C_19_H_22_N_2_O_7_, Mr = 390.38, crystal size = 0.21 × 0.44 × 0.77 mm3, monoclinic, space group C2/c, a = 22.7269(5) Å, b = 12.7839(2) Å, c = 13.7085(6) Å, β = 106.579(2)°, V = 3817.3(1) Å3, Z = 8, T = 130(2) K, 14040 reflections collected, 4450 unique reflections (Rint = 0.0163), R1 = 0.0360, wR2 = 0.0887 [I > 2σ(I)] and R1 = 0.0417, wR2 = 0.0929 [all data].

**4g**: C_21_H_24_N_2_O_7_, Mr = 416.42, crystal size = 0.17 x 0.23 x 0.79 mm3, monoclinic, space group I2/a, a = 17.7480(2) Å, b = 8.8899(1) Å, c = 27.1816(3) Å, β = 96.619(1)°, V = 4116.31(8) Å3, Z = 8, T = 130(2) K, 27541 reflections collected, 4942 unique reflections (Rint = 0.0202), R1 = 0.0435, wR2 = 0.1031 [I > 2σ(I)] and R1 = 0.0526, wR2 = 0.1094 [all data].

CCDC 1889259-1889260 contain the supplementary crystallographic data. These data can be obtained free of charge from The Cambridge Crystallographic Data Centre via www.ccdc.cam.ac.uk/data_request/cif.

### 3.3. Ultraviolet Spectroscopy

Spectra with a scan range of 290–400 nm were recorded in 50 or 25 μM methanol solutions, in 1 cm path length, 1.5 mL quartz cuvettes on a U-2800 double beam spectrophotometer (Hitachi, Tokyo, Japan) controlled by UV Solution version 2.2 software. The molar extinction coefficient at maximum absorption (ε_max_) of tested compounds was determined in methanol as the slope of the linear regression of absorbance vs. concentration of tested compound (from 10 to 50 μM or 2.5 to 30 μM).

### 3.4. Photoprotective Activity

#### 3.4.1. Cosmetic Formulations

Tested compounds and commercial UV filters as reference standards were incorporated to a neutral macrogel formulation in the concentration of 2%. Prior to the incorporation, compounds **3a–3e** and **4f–4h** and reference UV-filters were dissolved in a triacetin:Tween 20 (1:9) solvent system. For compounds **2a–2e** it was necessary to use DMSO. The final composition of the macrogol formulations is presented in the photoprotective activity section.

#### 3.4.2. In Vitro Photoprotection Study

In vitro photoprotection study was performed according to EN ISO 24443:2012 [71] with slight modifications. Formulation with tested compound (32.5 mg) was applied on a polymethylmethacrylate plate (PMMA) with the application area 25 cm^2^ and 5 µm roughness value to simulate the skin surface (Schonberg GmbH, Hamburg, Germany). As a reference a PMMA plate treated with approximately 15 µL of glycerin was used. Measurements were carried out by reflectance spectrophotometry with SPF-290AS Analyser (Solar Light Company, Glenside, PA, USA) equipped with integrating sphere controlled by WinSPF version X.X. software. For each sample two plates were prepared, absorbance measures were performed on 6 different positions of the plate from 290 to 400 nm with 1 nm steps. The results were expressed as an average of data from 12 scans. 

#### 3.4.3. Photostability Study

The photostability evaluation of compounds was performed both in methanol solutions and in macrogol formulations. Irradiation of samples according to previous studies [29,72] was conducted for 1 h at 500 W/m^2^ (cumulative dose of ultraviolet radiation 218 kJ/m^2^) with solar light simulator (Suntest CPS+, Atlas, Linsengericht, Germany) equipped with an optical filter cutting off wavelengths shorter than 290 nm and IR-block filter to neutralize thermal effects. The UV absorption spectrum of the samples were analysed post-irradiation and compared with pre-irradiation results. UPLC-MS/MS analyses were also performed.

Methanol solutions were tested at concentration 25 or 50 µM in quartz cuvettes with the PTFE stopper to prevent evaporation or leaking of the solvent. Macrogol formulations (2% *w/w*) were tested on PMMA plates (1.3 mg/cm^2^). After post-irradiation measure of UV absorption, exposed and unexposed plates were washed with 5 mL of methanol, then mixtures were filtered and analysed by UPLC-MS/MS. All analyses were performed in duplicates.

### 3.5. Skin Panel Model

Primary Epidermal Melanocytes (PCS-200-013, ATCC; Manassas, VA, USA), human epidermal keratinocyte cell line HaCaT (CLS Cell Lines Service; Eppelheim, Germany) and human skin fibroblast cell line BJ (CRL-2522, ATCC,) were used in the study. Primary Epidermal Melanocytes were cultured in Dermal Cell Basal Medium (ATCC) supplemented with Adult Melanocyte Growth Kit Components (ATCC), HaCaT and BJ cells were cultured in Dulbecco’s modified Eagle’s medium (DMEM; New York, NY, USA) and supplemented with 10% fetal bovine serum (FBS; Gibco, New York, NY, USA). Cell maintenance was performed in a humidified incubator at 37 °C with 5% CO_2_. Cells were passage when the culture has reached approximately 80 to 90% confluence. Cells were seeded at density of 6 × 10^3^ on 96-well plates. Following overnight culture, the cells were then treated with increasing doses of compounds and incubated for 24 h. Following cell exposure to each drug for 24 h, 10 µL MTT reagent (Sigma Aldrich, Darmstadt, Germany) was added to each well. After 4 hours incubation (37 °C, 5% CO_2_), the medium was aspirated and the formazan produced in the cells appeared as dark crystals in the bottom of the wells. Next, DMSO (dissolving solution) was added to each wells. Then the optical density (OD) of solution was determined at 570 nm on micro plate reader (Spectra iD3 Max, Molecular Devices; San Jose, CA, USA). Viability (% of control) was determined by dividing A_570_ of experimental wells by of A_570_ of control wells × 100%.

### 3.6. Hepatocytotoxicity and Neurocytotoxicity Studies

For hepatotoxicity and neurotoxicity assays a human neuroblastoma cell line—SH-SY5Y (CRL-2266™, ATCC) and a human hepatocellular carcinoma cell line–HepG2, respectively have been used. Cells were seeded on 96-well plates at an initial density of 10,000 cells per well. Cells were cultured in standard conditions for 24 h and then study compounds were administrated at different concentrations (10–50 μM) for another 24 h. After incubation time cells viability was measured with MTT assay. For this purpose culture medium has been replaced for the fresh one and MTT reagent (3-(4,5-dimethylthiazol-2-yl)-2,5-diphenyltetrazolium bromide) has been added at the concentration 0.5 mg/mL. Cells were incubated with MTT reagent at 37 °C for 4 h. Then formazan crystals have been dissolved in DMSO solution and the absorbance has been measured at 570 nm. Experiments were conducted in triplicates. Values represent mean percent of viability (in the percent of control).

### 3.7. Estrogenic Activity

To determine estrogenic activity of tested compounds their influence on estrogen-dependent MCF-7 breast cancer cell line proliferation was investigated. Cells were seeded into 24-wells plates in density of 40,000 per well. After 24 h incubation, cells were washed twice with PBS and fresh, estrogen- and phenol red-free medium (DMEM low-glucose; 2,5% Charcoal Stripped FBS; 2 mM glutamine; Sigma Aldrich, Darmstadt, Germany) was added. Than cells were incubated with tested compounds or benzophenone-2 (BP-2; Sigma Aldrich, Darmstadt, Germany), which is a UV filter exhibiting estrogenic activity, to final concentration of 5 μM. After 120 h incubations, cells proliferation was determined with crystal violet assay. Briefly, cells were washed with PBS and fixed with 3,7% formaldehyde. Then, crystal violet solution was added for 10 minutes. After incubation, solution was removed and cells were washed five times with PBS. Crystal violate was extracted from cells using destaining solution (1,33% citric acid, 1,09% sodium citrate in water/methanol (1:1) solution) and absorbance of the solution was determined at 570 nm (A_570_). Proliferation rate was determined by dividing the A_570_ of experimental wells by the A_570_ of control wells × 100%.

### 3.8. In Vitro Stability of Compounds in Mouse Liver Microsomes

Mouse liver microsomes (MLMs) were used to investigate the phase I metabolism of selected compounds (**2c, 2d**, **3b**, **4g**, **5a**) and the reference substance **BP-2**. Samples composed of test compound (20 µM), MLMs (0.8 mg/mL) and potassium phosphate buffer (100 mM, pH 7.4) were preincubated prior to addition of NADPH-regenerating system. NADPH-regenerating system contained NADP, glucose-6-phosphate, glucose-6-phosphate dehydrogenase and potassium phosphate buffer (100 mM, pH 7.4). Control incubations were performed in the absence of the NADPH-regenerating system [73] and in case of esters (**2c**, **2d**, **3b** and **4g**) without the mouse liver microsomes solution to determine spontaneous hydrolysis [59]. Incubations were conducted at 37 °C for at least 15 min up to 60 min. The reactions were stopped by the addition of 250 μL ice-cold methanol containing internal standard PTX (20 μM). The mixture was then centrifuged. Supernatant analysis was performed using UPLC/MS (Waters Corporation). The assays were repeated two times. The in vitro half times (t_1/2_) for test compounds were determined from the slope of the linear regression of ln % parent compound remaining versus incubation time. The calculated t_1/2_ was incorporated into the following equation to obtain intrinsic clearance: (Cl_int_) = (volume of incubation [µL]/ protein in the incubation [mg]) × 0.693 / t_1/2_ [74].

### 3.9. Ames Assay

The assay is a liquid microplate modification of the traditional *Salmonella* test [75,76]. During the procedure, bacteria are exposed to different concentrations of a test compound for 90 min in a medium containing sufficient histidine (*S. typhimurium*) or tryptophan (*E. coli*) to support cell divisions. Then, the cultures are diluted in pH indicator medium lacking histidine or tryptophan and aliquoted into 48 wells of a 384-well plate. Within 48 hours, cells that have undergone reversion to amino acid prototrophy will grow into colonies. Bacterial metabolism reduces pH of the medium, changing the color of that well from purple to yellow (https://www.xenometrix.ch [77]).

The following strains were used in the study: *S*. *typhimurium* TA98, TA100, TA1535, TA1537 and *E*. *coli* wp2 uvrA[pKM101]. TA100, TA1535 and *E*. *coli* strains are used for the detection of base substitution mutations, whereas TA98 and TA1537 are utilized for the detection of frameshift mutations. *S*. *typhimurium* strains have GC base pairs, whereas *E*. *coli* strain has AT base pair at their primary reversion site and detect certain oxidizing mutagens, cross-linking agents and hydrazines.

The test procedure provided by Xenometrix (https://www.xenometrix.ch [77]) and described earlier was followed [78,79,80]. Briefly, the bacterial strains were grown overnight in exposure medium and exposed to test compounds **4g** and **3b** in 24-well plates for 90 min at 37 °C with agitation in the absence (–S9) or presence (+S9) of 4.5% phenobarbital/ β-naphthoflavone-induced rat liver S9. Compounds **4g** and **3b** were dissolved in DMSO and tested at final concentrations of 0.1, 0.2 and 0.5 mM. After the preincubation, the cultures were diluted in the indicator medium and the contents of each 24-well culture were distributed into 48 wells on a 384-well plate and incubated further for 48 h at 37 °C without agitation. After the exposure, the number of wells containing bacteria was scored by eye for yellow wells. Positive and negative controls were included in the assay, and all doses were done in triplicate.

Positive controls used for the MPF protocol were 2-nitrofluorene (2-NF) at 2 µg/mL (TA98, –S9); 4-nitroquinoline-N-oxide (4-NQO) at 0.1 µg/mL (TA100, –S9); N4-aminocytidine (N4-ACT) at 100 µg/mL (TA1535, –S9); 9-aminoacridine (9-AAc) at 15 µg/mL (TA1537, -S9); 4-NQO at 2 µg/mL (*E.coli* uvrA[pKM101], –S9); 2-aminoanthracene (2-AA) at 0.5 µg/mL (TA98, + S9); 2-AA at 1.25 µg/mL (TA100, + S9); 9-AA at 2.5 µg/mL (TA1535 and TA1537, + S9); 2-aminofluorene (2-AF) at 400 µg/mL (*E.coli* uvrA[pKM101], + S9). Pure DMSO was used as the negative control.

To evaluate the test results the following criteria were used: the fold increase in the number of positive wells over the solvent control baseline (FIB), and the dose dependency. The fold increase of revertants relative to the solvent control was determined by dividing the mean number of positive wells at each dose by the solvent control at baseline. The solvent control at baseline was defined as the mean number of positive wells in the solvent control plus one standard deviation (SD). When an increase of more than 2-fold relative to the baseline at more than one dose with a dose–response is observed the sample is classified as positive, whereas when no response >2 times the baseline and no dose–response is stated the sample is negative [76,77,78,79,80].

## 4. Conclusions

In summary all tested compounds may be considered as potential UVA or UVB filters. Their absorption parameters are comparable or more favorable than those of selected commercially available UV-filters. Compound **4f** (diethyl 2,2′-((*Z*)-2,4-dioxo-5-((*E*)-3-phenylallylidene)-imidazolidyne-1,3-diyl)diacetate) shows the best UVA photoprotective properties among the tested series with a UVA PF of 8.43 ± 1.35 but its photostability is unsatisfactory. Compound **2a** (ethyl (*Z*)-2-(4-benzylidene-2,5-dioxoimidazolidin-1-yl)acetate) shows the best UVB photoprotective properties within the tested series with a SPF*_in vitro_* of 4.79 ± 0.02, but it is only slightly soluble in the formulations used. Considering the other features of modern UV-filters such as good solubility in cosmetic solvents and high photostability, the most favorable UVA-filter seems to be compound **4g** (diethyl 2,2′-((*Z*)-4-((*E*)-3-(4-methoxyphenyl)allylidene)-2,5-dioxoimidazolidine-1,3-diyl)diacetate) with a UVA PF of 6.83 ± 0.05 and better photostability than compound **4f**. The most promising potential UVB-filter is compound **3b** (diethyl 2,2′-((*Z*)-4-(4-methoxybenzylidene)-2,5-dioxoimidazolidine-1,3-diyl)- diacetate) with SPF*_in vitro_* of 3.07 ± 0.04, characterized by very good solubility and photostability. The preliminary safety assessment of the compounds is also promising. Tested compounds at high concentrations (50 µM) are non-toxic toward human skin cells, hepatoma and neuroblastoma cells and possess no estrogenic activity. We may conclude that neither test compounds **3b** and **4g** nor their potential metabolites are base substitution or frameshift mutagens. Their metabolic stability is various and depends on substituents in phenyl and imidazolidine ring.

## Figures and Tables

**Table 1:** Ultraviolet spectroscopic properties of the tested 5-arylideneimidazolidine-2,4-dione derivatives and reference UV filters obtained in methanol solutions.

Compound	Chemical Structure	λ_max_ [nm]	ε_max_ [M^−1^ cm^−1^]	Absorption Range [nm]
**1e**	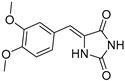	341	28440	290–384
**2a**	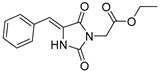	316	28460	290–345
**2b**	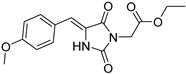	334	31920	290–372
**2c**	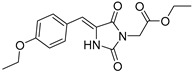	336	35610	290–376
**2d**	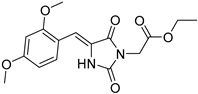	349	30700	290–394
**2e**	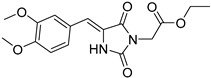	344	30740	290–381
**3a**	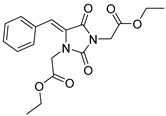	295	13980	290–335
**3b**	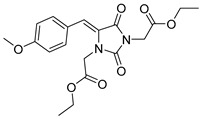	318	16310	290–362
**3c**	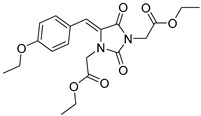	321	17010	290–367
**3d**	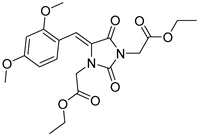	333	17310	290–374
**3e**	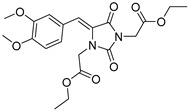	329	14200	290–380
**4f**	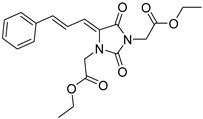	354	41477	295–389
**4g**	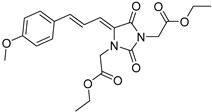	379	54996	290–400
**4h**	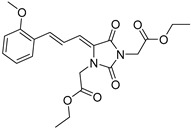	370	34987	290–400
**5a**	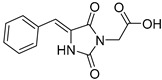	316	27310	290–346
**4-MBC**	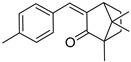	299	25550	290–332
**Octocrylene**	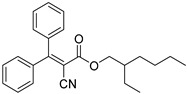	302	13650	290–349
**EHMC**	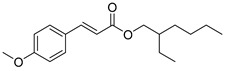	309	22990	290–339
**Avobenzone**	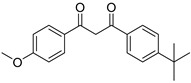	358	36820	290–393

**Table 2:** Photoprotective activity of tested compounds and reference UV-filters obtained in 2% (*w/w*) macrogel formulations (F1^*^, F2^#^, F3^^^).

Compound	Chemical Structure	SPF*_in vitro_*	UVA PF	λ_c_	UVA/UVB Ratio
**2a^#^**	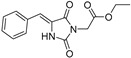	4.79 ± 0.02	2.40 ± 0.01	339	0.35 ± 0.00
**2b^#^**	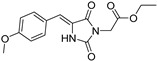	3.20 ± 0.07	4.29 ± 0.11	363	1.20 ± 0.00
**2c^^^**	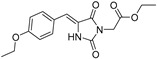	3.16 ± 0.05	4.68 ± 0.02	367	1.36 ± 0.01
**2d^^^**	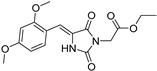	2.00 ± 0.11	4.48 ± 0.38	386	2.59 ± 0.09
**2e^^^**	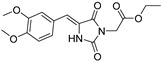	2.50 ± 0.08	4.77 ± 0.24	379	1.95 ± 0.02
3a^*^	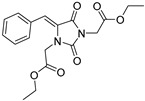	2.97 ± 0.45	1.29 ± 0.09	331	0.16 ± 0.02
3b^*^	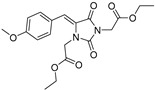	3.07 ± 0.04	2.59 ± 0.23	366	0.84 ± 0.08
3c^*^	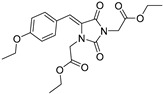	2.49 ± 0.08	2.46 ± 0.01	370	1.05 ± 0.03
3d^*^	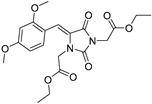	1.84 ± 0.02	2.35 ± 0.03	383	1.63 ± 0.01
3e^*^	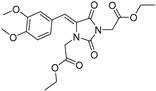	1.87 ± 0.08	2.17 ± 0.11	382	1.43 ± 0.02
4f^*^	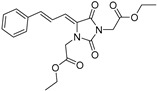	2.51 ± 0.17	8.43 ± 1.35	381	2.58 ± 0.06
4g^*^	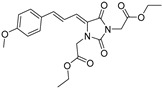	1.68 ± 0.04	6.83 ± 0.05	391	4.50 ± 0.32
4h^*^	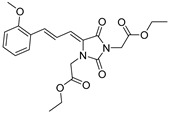	1.94 ± 0.04	4.28 ± 0.02	389	2.58 ± 0.01
Octocrylene^*^	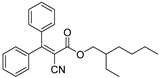	2.87 ± 0.16	1.20 ± 0.03	327	0.12 ± 0.01
EHMC^*^	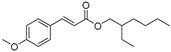	4.96 ± 0.33	1.34 ± 0.05	321	0.05 ± 0.01
4-MBC^*^	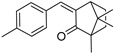	5.59 ± 0.27	1.08 ± 0.1	316	0.00 ± 0.00
Avobenzone^*^	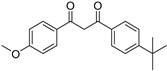	2.23 ± 0.03	7.56 ± 0.19	381	2.49 ± 0.03

Results are presented as mean ± SD from two independent experiments, at each determination six scans were performed. ^*^F1–Tween 20 43.2%, PEG-400 25%, PEG-1500 25%, Triacetin 4.8%, tested compound 2%; ^#^F2–Tween 20 43.2%, PEG-400 25%, PEG-1500 25%, DMSO 4.8%, tested compound 2%; ^^^F3–PEG-400 25%, PEG-1500 25%, DMSO 48%, tested compound 2%.

**Table 3:** The changes in photoprotective activity of tested compounds and reference UV-filters after irradiation with solar light simulator at 500 W/m^2^. In the experiments appropriate formulations (F1^*^, F2^#^, F3^^^) were used.

Compound	% of Initial SPF*_in vitro_*	% of Initial UVA PF	% of Initial AUC
**2a^^^**	33.25	37.87	46.72
**2a^#^**	55.49	68.96	62.42
**2b^^^**	36.51	24.13	35.28
**2b^#^**	57.19	54.31	59.55
**2c^^^**	44.37	35.83	34.13
**2d^^^**	75.69	51.45	58.87
**2e^^^**	56.00	38.99	41.99
3a^*^	83.31	103.50	88.19
3b^*^	80.46	87.04	84.67
3b^*^	72.69	84.08	76.50
3c^*^	88.73	86.59	85.29
3d^*^	89.92	97.66	94.01
3e^*^	81.28	87.10	79.59
4f^*^	62.24	25.98	27.92
4g^*^	86.29	34.39	51.98
4h^*^	82.22	62.38	69.19
4-MBC^*^	91.05	101.39	90.89
EHMC^*^	80.83	88.76	78.45

Results are presented as means from two independent experiments, at each determination six scans were performed. ^*^F1–Tween 20 43.2%, PEG-400 25%, PEG-1500 25%, Triacetin 4.8%, tested compound 2%; ^#^F2–Tween 20 43.2%, PEG-400 25%, PEG-1500 25%, DMSO 4.8%, tested compound 2%; ^^^F3–PEG-400 25%, PEG-1500 25%, DMSO 48%, tested compound 2%.

**Table 4:** In vitro stability (t_1/2_ and Cl_int_) of compounds **1e, 2c**–**2e, 3b, 4g, 5a** and reference substance benzophenone-2 (**BP-2**) in mouse liver microsomal system. Metabolites formation in the presence and absence of NADPH; compounds elemental composition changes and probable transformation pathways.

Compound	[M + H^+^] Da	t _½_ (min)	*Cl_int_* (µL/mg/min)	Metabolite Formation in the Presence (P) or/and Absence (A) of NADPH	Elemental Composition Change	Probable Transformation
**1e**	249	61	14	M1 P	−14	*O*-dealkylation
**2c**	319	33	52	M1 ^a^ P	−28	*O*-dealkylation
**2d**	335	15	59	M1^a^ P	−14	*O*-dealkylation
**2e**	335	41	21	M1 P	−14	*O*-dealkylation
**3b**	391	12	75	M1 P and A M2 P	−28 −14	hydrolysis *O*-dealkylation
**4g**	417	3	328	M1 P and A M2 P	−28 −14	hydrolysis *O*-dealkylation
**5a**	247	1386	0.6	ND		
**BP-2**	247	66	13	NT	NT	NT

ND–not detected; NT–not tested.

## Supplementary Materials

The following are available online at https://www.mdpi.com/1420-3049/24/12/2321/s1, Figure S1: The distribution of the angle values between the planes of hydantoin and aromatic rings in crystal structures containing a 5-benzylidenehydantoin (hyd), 5-benzylidene-2-thiohydantoin (S-hyd) or 5-benzylidene-2-selenohydantoin (Se-hyd) fragment retrieved from the CSD. Figure S2: The overlap of hydantoin rings of **3b** (carbon atoms in grey) and **4g** (carbon atoms in green). The disordered fragment of **3b** is depicted only for major occupancy (**A**). Figure S3: UV-absorption spectra of tested compounds and reference UV filters obtained in methanol solutions (for **2a–2e**, **3a**–**3e**, octocrylene and EHMC at 50 µM, for **4f**–**4h** and avobenzone at 25 µM). Figure S4: UV absorption spectra of tested compounds and EHMC obtained pre-irradiation and 1 hour after irradiation with solar light simulator conducted at 500 W/m2 in 25 µM (4g) or 50 µM (2d, 3b, EHMC) methanol solutions. Figure S5: The chromatograms and mass spectra of methanol solutions of compounds **3b** and **4g** pre-irradiation (**A**) and 1 hour after irradiation (**B**) with solar light simulator conducted at 500 W/m2. Table S1: Parameters of intermolecular interactions in the crystal structures of **3b** and **4g**. Table S2: UV absorption changes after 1h irradiation at 500 W/m^2^ of methanol solution of tested compounds and reference UV-filters. Table S3: Mutagenic activity of compounds **4g** and **3b** tested with the Ames assay.
