# Supplementary material for: Discovery of Novel UV-Filters with Favorable Safety Profiles in the 5-Arylideneimidazolidine-2,4-dione Derivatives Group
Source: Molecules. 2019 Jun 24;24(12):2321. doi: 10.3390/molecules24122321 (PMC6630718; doi:10.3390/molecules24122321)
Supplement: Supplementary file 1 [file molecules-24-02321-s001.pdf]

Discovery of Novel UV-Filters with Favorable Safety Profiles in the 5-Arylideneimidazolidine-2,4-dione Derivatives Group

Justyna Popiół, Agnieszka Gunia-Krzyżak, Kamil Piska, Dorota Żelaszczyk, Paulina Koczurkiewicz, Karolina Słoczyńska, Katarzyna Wójcik-Pszczoła, Anna Krupa, Agata Kryczyk-Poprawa, Ewa Żesławska, Wojciech Nitek, Paweł Żmudzki, Henryk Marona, Elżbieta Pękala

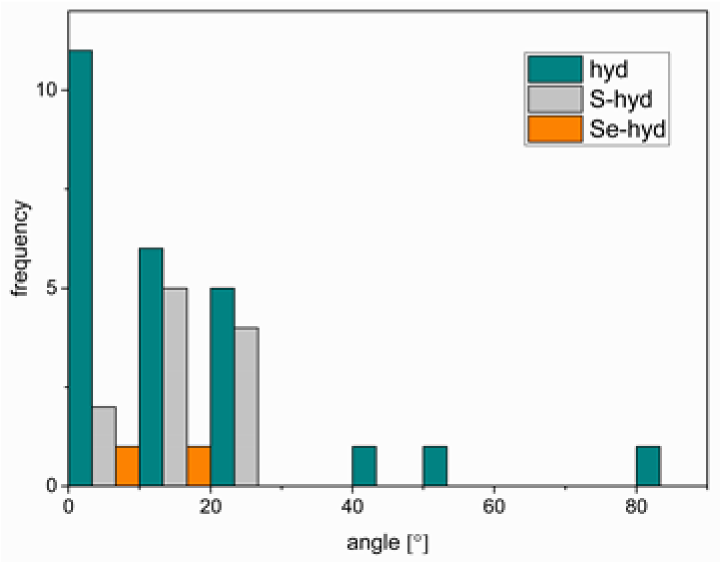

Figure S1. The distribution of the angle values between the planes of hydantoin and aromatic rings in crystal structures containing a 5-benzylidenehydantoin (hyd), 5-benzylidene-2-thiohydantoin (S-hyd) or 5-benzylidene-2-selenohydantoin (Se-hyd) fragment retrieved from the CSD.

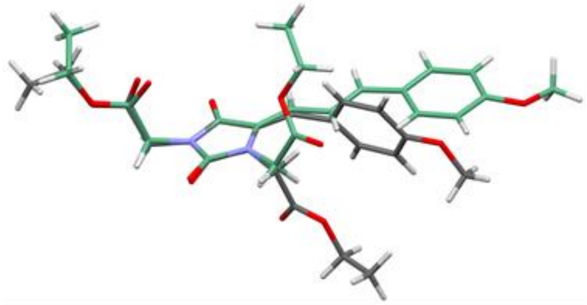

Figure S2. The overlap of hydantoin rings of **3b** (carbon atoms in grey) and **4g** (carbon atoms in green). The disordered fragment of **3b** is depicted only for major occupancy (**A**).

Table S1. Parameters of intermolecular interactions in the crystal structures of **3b** and **4g**

	D-H···A	H···A (Å)	D···A (Å)	D-H-A (°)	Symmetry codes
**3b**	C8-H8A···O4	2.56	3.519(2)	163	−x + 1, −y + 2, −z + 2
	C8-H8B···O4	2.64	3.400(2)	134	x, −y + 2, z + ½
	C9-H9A···O6A	2.52	3.415(5)	152	x, y + 1, z
	C10-H10A···O2	2.29	3.130(2)	142	−x + 1/2, −y + 3/2, −z + 2
	C14-H14···O1	2.76	3.354(1)	121	−x + 1, −y + 2, −z + 2
	C16-H16···O1	2.63	3.383(2)	137	−x + 1, −y + 2, −z + 2
	C21-H21B···O4	2.54	3.495(2)	165	x, y + 1, z
**4g**	C6-H6A···O7	2.64	3.328(2)	127	−x + 1/2, y−1, −z
	C6-H6B···O1	2.59	3.399(2)	139	−x + 1/2, y, −z
	C13-H13C···O6	2.54	3.470(2)	160	−x, y + 1/2, −z + ½
	C14-H14···O4	2.22	2.965(2)	134	−x + 1/2, −y + 3/2, z
	C16-H16···O4	1.68	2.586(2)	158	−x + 1/2, −y + 3/2, z
	C19-H19···O2	2.49	3.425(2)	168	−x + 1/2, y + 1/2, −z
	C23-H23C···O5	2.65	3.310(2)	125	x + 1/2, −y + 2, z

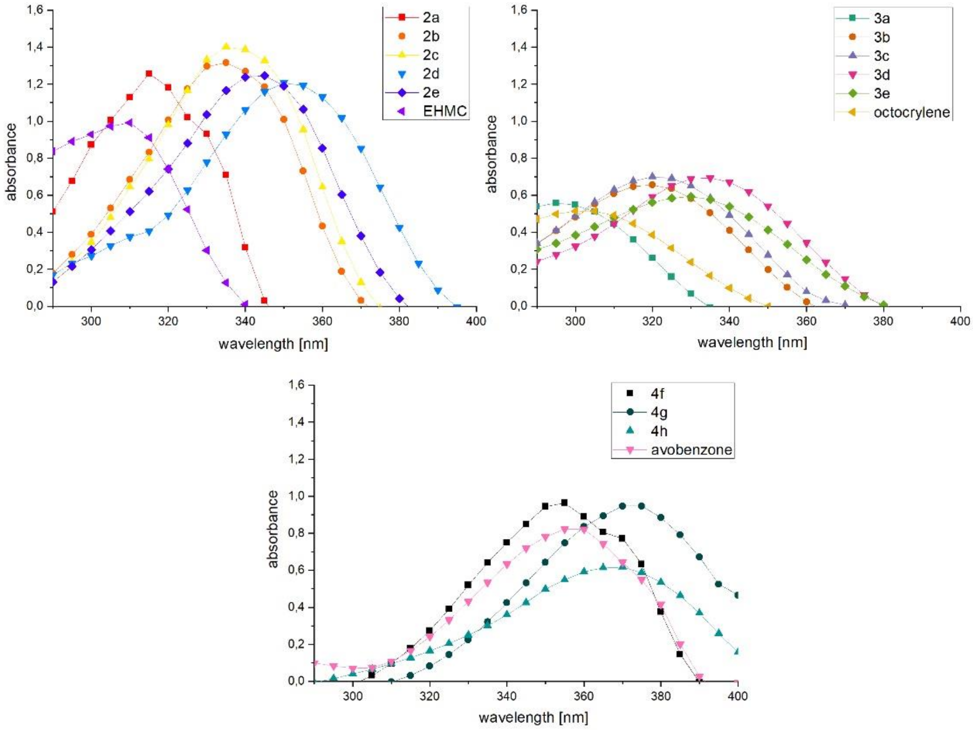

Figure S3. UV-absorption spectra of tested compounds and reference UV filters obtained in methanolic solutions (for **2a–2e**, **3a–3e**, octocrylene and EHMC at 50 μM, for **4f–4h** and avobenzone at 25 μM).

Table S2. UV absorption changes after 1h irradiation at 500 W/m^2 of methanol solution of tested compounds and reference UV-filters.

Compd	λ_{max} pre-irradiation	λ_{max} post-irradiation	% of initial AUC post-irradiation
**1e**	341	346	95.38
**2a**	316	317	70.45
**2b**	334	341	95.67
**2c**	336	344	96.89
**2d**	349	354	100.96
**2e**	344	351	97.8
**3a**	295	301	122.34
**3b**	318	326	114.39
**3c**	321	329	119.42
**3d**	333	342	111.55
**3e**	329	340	129.32
**4f**	354	354	59.12
**4g**	379	378	80.15
**4h**	370	370	98.55
4-MBC	299	303	87.76
EHMC	309	307	63.26

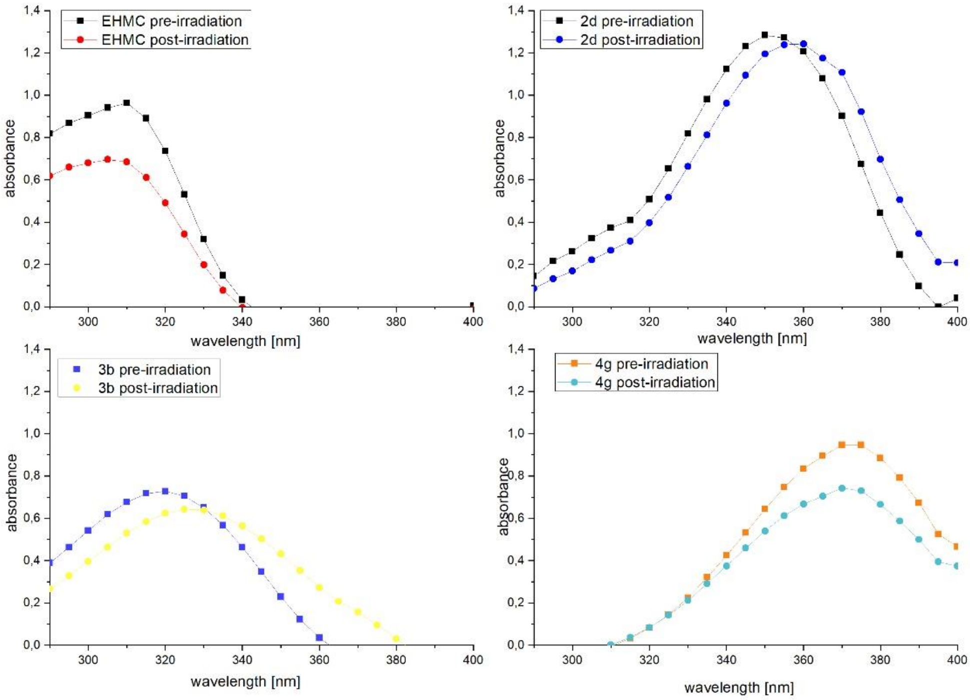

Figure S4. UV absorption spectra of tested compounds and EHMC obtained pre-irradiation and 1 hour after irradiation with solar light simulator conducted at 500 W/m^2 in 25 μM (**4g**) or 50 μM (**2d**, **3b**, EHMC) methanol solutions.

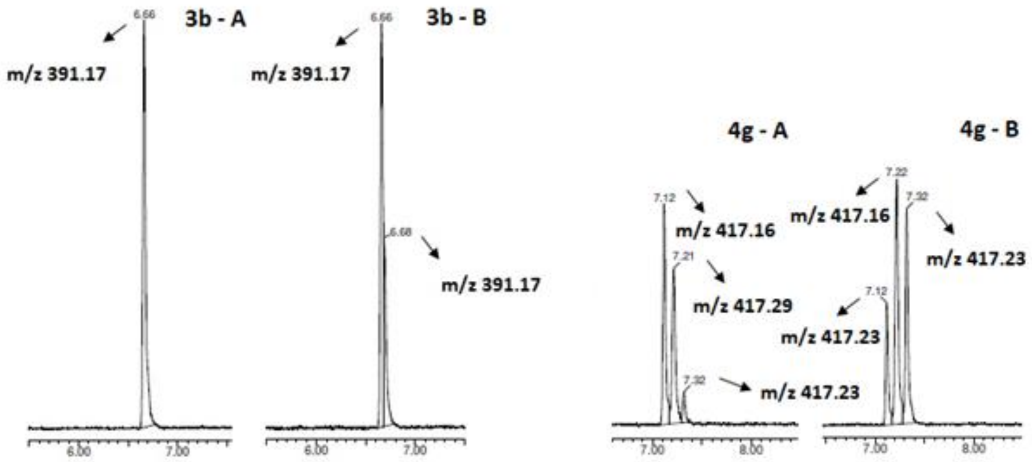

Figure S5. The chromatograms and mass spectra of methanol solutions of compounds **3b** and **4g** pre-irradiation (**A**) and 1 hour after irradiation (**B**) with solar light simulator conducted at 500 W/m^2.

Table S3. Mutagenic activity of compounds **4g** and **3b** tested with the Ames assay.

		**Positive wells per microplate**									
		S. typhimurium								***E. coli***	
		**TA98**		**TA100**		**TA1535**		**TA1537**		**WP2**	
		**–S9**	**+S9**	**–S9**	**+S9**	**–S9**	**+S9**	**–S9**	**+S9**	**–S9**	**+S9**
**Compound**	**Conc. (mM)**	**FIB***									
**4g**	0.1	0.5	0.3	1.1	0.7	0.3	0.3	0.3	1.4	0.3	0.6
	0.2	0.9	0.4	0.3	0.5	1.4	0.7	1.7	1.1	1.3	1.0
	0.5	0.2	0.6	0.6	0.5	0.6	0.3	0.5	0.8	1.3	0.7
	PC**	25.3	12.6	4.3	3.1	40.0	16.5	48.0	38.3	25.7	4.0
**3b**	0.1	0.9	0.2	1.1	0.7	0.8	0.5	0.3	1.1	0.3	0.7
	0.2	0.2	0.4	0.8	0.6	0.6	0.1	0.3	1.1	0.7	0.6
	0.5	0.9	0.3	0.7	0.6	1.7	0.1	0.3	0.3	0.3	0.4
	PC**	25.3	12.6	4.3	3.1	40.0	16.5	48.0	38.3	25.7	4.0

*FIB–fold induction over baseline (baseline = mean zero-dose control + 1 SD); SD–standard deviation
**Positive controls: 2-Nitrofluorene (2-NF) at 2 μg/mL (TA98, –S9); 4-Nitroquinoline-*N*-oxide (4-NQO) at 0.1 μg/mL (TA100, –S9); N4-Aminocytidine (N4-ACT) at 100 μg/mL (TA1535, –S9); 9-Aminoacridine (9-AAc) at 15 μg/mL (TA1537, –S9); 4-NQO at 2 μg/mL (*E.coli uvrA*[pKM101], –S9); 2-Aminoanthracene (2-AA) at 0.5 μg/mL (TA98, +S9); 2-AA at 1.25 μg/mL (TA100, +S9); 9-AA at 2.5 μg/mL (TA1535 and TA1537, +S9); 2-Aminofluorene (2-AF) at 400 μg/mL (*E.coli uvrA*[pKM101], +S9).
